# Standardized Extract of Atractylodis Rhizoma Alba and Fructus Schisandrae Ameliorates Coughing and Increases Expectoration of Phlegm

**DOI:** 10.3390/molecules25133064

**Published:** 2020-07-04

**Authors:** Hee-Sung Chae, Sun Young Kim, Pisey Pel, Jungmoo Huh, Sun-Woo Joo, Yun Young Lim, Shin Jung Park, Jong Lae Lim, Young-Won Chin

**Affiliations:** 1 College of Pharmacy and Research Institute of Pharmaceutical Sciences, Seoul National University, 1, Gwanak-ro, Gwanak-gu, Seoul 08826, Korea; chaeheesung83@gmail.com (H.-S.C.); pisey.1603@gmail.com (P.P.); jmhuh112@gmail.com (J.H.); 2 Department of Botanical Drug, Chong Kun Dang (CKD) Pharm Research Institute, Yongin-si, Gyeonggi-do 16995, Korea; syk@ckdpharm.com (S.Y.K.); sunwoo@ckdpharm.com (S.-W.J.); yylim@ckdpharm.com (Y.Y.L.); parksj@ckdpharm.com (S.J.P.); jllim@ckdpharm.com (J.L.L.)

**Keywords:** Atractylodis Rhizoma Alba, Fructus Schisandrae, cough, phlegm, interleukin-8, TNF-α, TRPV-1, mucociliary clearance, CKD-497

## Abstract

Cough and phlegm frequently occur in respiratory diseases like upper respiratory tract infections, acute bronchitis, and chronic obstructive pulmonary diseases. To relieve these symptoms and diseases, various ingredients are being used despite the debates on their clinical efficacy. We aimed to investigate the effects of the extract CKD-497, composed of Atractylodis Rhizoma Alba and Fructus Schisandrae, in relieving cough and facilitating expectoration of phlegm. CKD-497 was found to inhibit inflammatory mediators such as interleukin-8 (IL-8) and tumor necrosis factor α (TNF-α) in lipopolysaccharide (LPS)-treated mouse macrophages and transient receptor potential cation channel 1 (TRPV-1)-overexpressed human bronchial epithelial cells stimulated by capsaicin. CKD-497 decreased the viscosity of the mucin solution. During in vivo experiments, CKD-497 reduced coughing numbers and increased expectoration of phlegm via mucociliary clearance enhancement. Collectively, these data suggest that CKD-497 possesses potential for cough and phlegm expectoration treatment.

## 1. Introduction

Cough and sputum are the common symptoms of acute upper respiratory tract infection (AUPTI), acute bronchitis (AB), and chronic obstructive pulmonary disease (COPD) [1,2]. Both cough and the production of phlegm have been recognized as defense mechanisms in the respiratory system. In the normal condition, a cough is known as a protective respiratory reflex to remove particularly airway mucus that traps foreign substances and prevents them from falling deeper into the respiratory system [3,4]. Airway mucus, secreted from epithelial cells and submucosal glands in the airway, is mobile and viscous, but well-cleared by cough and ciliary movement. However, dysfunctions in airway mucin secretion and/or mucus hydration produce excessively viscous mucus that is not easily cleared by cough and ciliary action. A variety of stimuli including viruses and some cytokines increase hyperproduction of mucins like MUC5AC and MUC5B, which are controlled by interleukin-13 and interleukin-1β [4]. The inflammatory responses in the airway are associated with the production of phlegm, airway mucus-containing bacteria, inflammatory cells, and inflammatory mediators, which cause severe coughing and purulent airway mucus. To alleviate severe coughing and phlegm, several antitussives and expectorants such as ivy extract preparation [5], theobromine, and ambroxol are being used, but their clinical usefulness for AUPTI, AB, and COPD is controversial due to their therapeutic outcomes [6,7,8]. Despite the controversy, the sales of over-the-counter cough and phlegm medicines in the international market have expanded to a one billion euro market [9,10].

Mucin secretion and inflammation in the airway epithelial cells are also mediated by TRPV1 activity, which is involved in the epidermal growth factor receptor (EGFR)/ phosphatidylinositol 3-kinases (PI3K) and hypoxia-inducible factors (HIF)-1α/ protein kinase C (PKC) pathways [11]. Activation of TRPV1 in the airway epithelium was implicated in the pathogenesis of persistent cough and COPD [12]. Although TRPV1 has been localized in the epidermis, little is known about the physiological significance and functional role of TRPV1. Activation of epidermal TRPV1 induces the expression of nitric oxide and the release of pro-inflammatory mediators such as interleukins, transforming growth factor-β (TGF-β), and tumor necrosis factor-α (TNF-α) [13]. In this study, we observed that TRPV1 mediates cytokine expression in Raw 264.7 and BEAS-2B cells. Herein, the new proprietary formula CKD-497, composed of Atractylodis Rhizoma Alba and Fructus Schisandrae, was investigated for its alleviating activities against cough and increasing expectorant activity in vitro and in vivo. 

## 2. Results

### 2.1. Isolation and Quantitative Analysis

**Figure 1:**
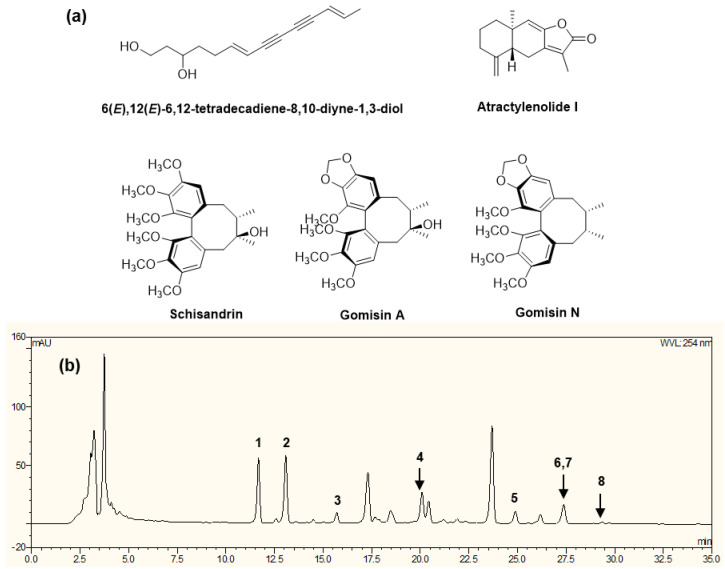
Isolated compounds (**a**) and HPLC-UV analysis of CKD-497 (**b**) **1.** 6(*E*),12(*E*)-tetradecadiene-8,10-diyne-1,3-diol, **2.** schisandrin, **3.** gomisin A, **4.** atractylenolide I, **5.** schisandrin A, **6.** gomisin N, **7.** γ-schisandrin, **8.** schisandrin C.

The main components for the extract of Atractylodis Rhizoma Alba and Fructus Schisandrae were isolated, which led to the isolation and identification of five compounds: schisandrin, gomisin A, atractylenolide I, gomisin N, and 6(*E*),12(*E*)-tetradecadiene-8,10-diyne-1,3-diol. Along with the isolated compounds, three compounds (schisandrin A, schisandrin C, and γ-schisandrin) obtained from the in-house chemical library were used for high-performance liquid chromatography-ultraviolet (HPLC-UV) analysis for CKD-497, as shown in Figure 1, suggesting that schisandrin, gomisin A, atractylenolide I, gomisin N, 6(*E*),12(*E*)-tetradecadiene-8,10-diyne-1,3-diol, schisandrin A, schisandrin C, and γ-schisandrin were present in the current extract, CKD-497 [14,15]. To control the quality of the extract used in the present study, quantitative analysis of the isolated compounds was performed, and the results confirmed the existence of compounds 6(*E*),12(*E*)-tetradecadiene-8,10-diyne-1,3-diol: 1.096 ± 0.140 mg/g; schisandrin: 1.904 ± 0.197 mg/g; gomisin A: 0.271 ± 0.042 mg/g; gomisin N: 1.139 ± 0.115 mg/g; and atractylenolide I: 0.875 ± 0.092 mg/g, respectively.

### 2.2. The Effects of CKD-497 on LPS-Induced NO and Cytokine Production in Raw 264.7 Cells

**Figure 2:**
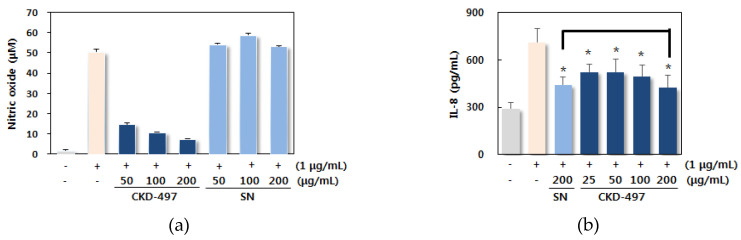

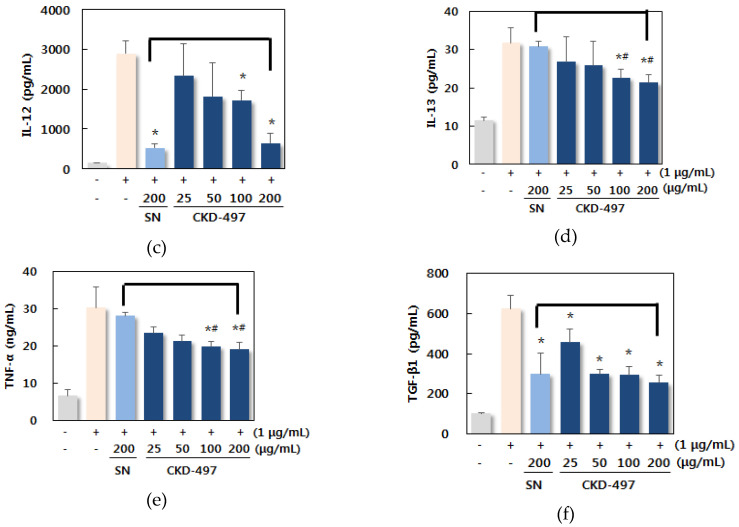
CKD-497 reduced cytokines in Raw 264.7 cells. Supernatant of cells after CKD-497 treatment was harvested. The level of (**a**) Nitric oxide (NO) assay was conducted and (**b**) interleukin 8 (IL-8) (**c**) IL-12 (**d**) IL-13 (**e**) tumor necrosis factor α (TNF-α) and (**f**) transforming growth factor-β1 (TGF-β1) was measured by ELISA in accordance with manufacturer’s instructions. Synatura was used as a positive control. All experiments were repeated three times and data were expressed as the mean ± standard error of the mean (SEM) (*n* = 3). * *p* < 0.05 compared with the induced group [lipopolysaccharide (LPS)-treated group]. ^#^ *p* < 0.05 compared with the Synatura (SN) group.

To determine the anti-inflammatory effect of CKD-497, we investigated whether CKD-497 reduces nitric oxide (NO) and cytokines in Raw 264.7 cells. Lipopolysaccharide (LPS) (1 μg/mL) induced NO in the media of Raw 264.7 cells, which was significantly reduced by adding CKD-497 (50, 100, 200 μg/mL) in a concentration-dependent manner, whereas Synatura (SN) did not (Figure 2a). CKD-497 at both 100 and 200 μg/mL significantly reduced the expression of IL-13 and TNF-α, whereas lower concentrations of CKD-497 and SN did not show significant effectiveness (Figure 2d,e). CKD-497 at 200 μg/mL ameliorated expression of IL-8, IL-12, and TGF-β1 at the same level as SN (Figure 2b,c,f). CKD-497 was able to inhibit production or release of NO, IL-8, IL-12, IL-13, TNF-α, and TGF-β1.

### 2.3. The Effects of CKD-497 on Capsaicin-Induced TRPV-1 and Cytokine Production in BEAS-2B Cells

**Figure 3:**
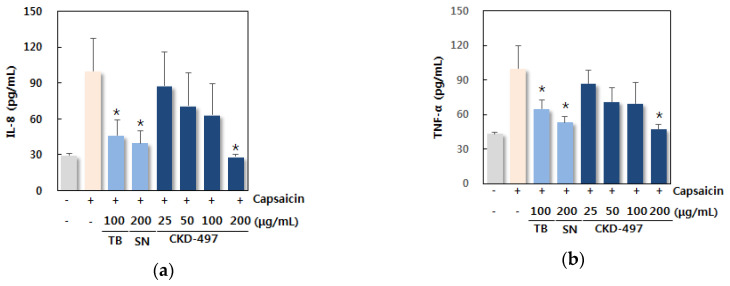

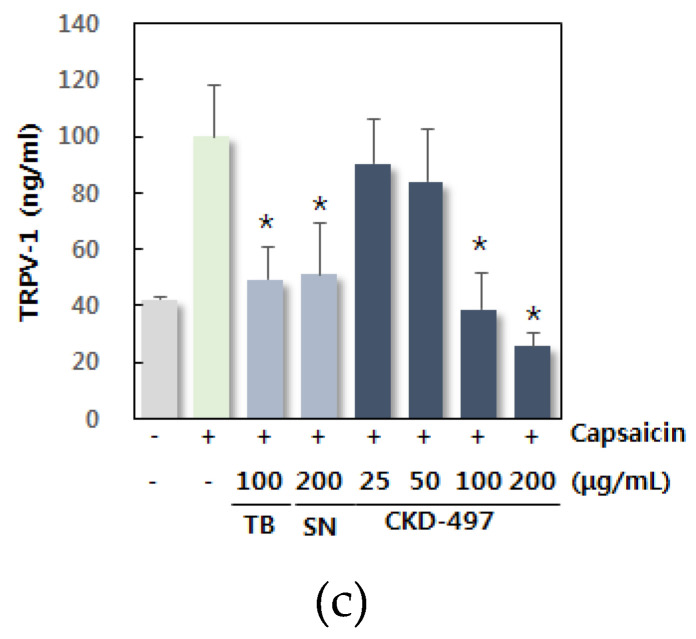
CKD-497 reduced cytokines in TRPV-1-overexpressed BEAS-2B human bronchial epithelial cell. Supernatant of cells after CKD-497 treatment was harvested. The levels of (**a**) IL-8, (**b**) TNF-α, and (**c**) TRPV-1 were measured by ELISA in accordance with the manufacturer’s instructions. Theobromine (TB) and Synatura (SN) were used as positive controls. All experiments were repeated three times and data are expressed as the mean ± SEM (*n* = 3). * *p* < 0.05 compared with the induced group (capsaicin-treated group).

We also determined the effect of CKD-497 on cytokines in TRPV-1-overexpressed BEAS-2B human bronchial epithelial cells. Capsaicin was used as inducer [16], an agonist of TRPV-1 receptor in coughing. The expression of TRPV-1 was decreased by CKD-497 at 100 and 200 μg/mL, which was more significant than theobromine and SN (Figure 3a). CKD-497 at 200 μg/mL reduced IL-8 and TNF-α as theobromine and SN (Figure 3b,c). These data suggested that CKD-497 has an inhibitory effect on TRPV-1 expression and cytokines.

### 2.4. The Effects of CKD-497 on Viscosity

**Figure 4:**
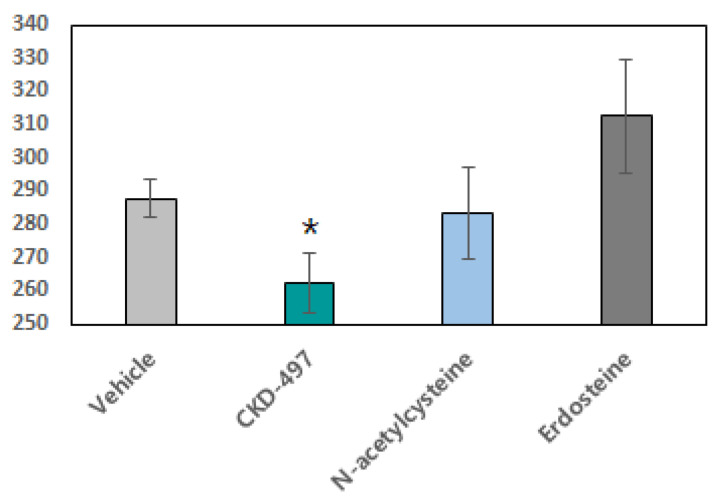
CKD-497 reduced viscosity. CKD-497 and two positive control (*N*-acetylcysteine, erdosteine) were added in 15% of mucin solution and then stirred at 37 ℃ for 1 h. *N*-acetylcysteine and erdosteine viscosity was detected by microrheometer. All experiments were repeated three times and data are expressed as the mean ± SEM (*n* = 3). * *p* < 0.05 compared with vehicle.

Increases in mucus viscosity and elasticity contribute to mucostasis and its pathophysiological consequences. Alterations in these parameters by sulfhydryl-reactive agents such as *N*-acetylcysteine are thought to be the primary mechanism for the improved mucociliary clearance of these agents. Therefore, we examined the effects of the three drugs on mucus viscosity. To demonstrate whether CKD-497 reduced mucin viscosity, we made a 15% mucin solution and detected viscosity after addition of CKD-497, *N*-acetylcysteine, and erdosteine. However, there were no significant changes in the parameters from either erdosteine or *N*-acetylcysteine at same concentration of CKD-497. As shown in Figure 4, CKD-497 showed a more significant reduction (262.3 ± 8.9 mPa·s) of mucin viscosity than either of the other drugs (283.4 ± 13.6 mPa·s and 312.5 ± 17.3 mPa·s for *N*-acetylcysteine and erdosteine, respectively).

### 2.5. The Effects of CKD-497 on Cough Frequency and Expectorant Activity In Vivo

**Figure 5:**
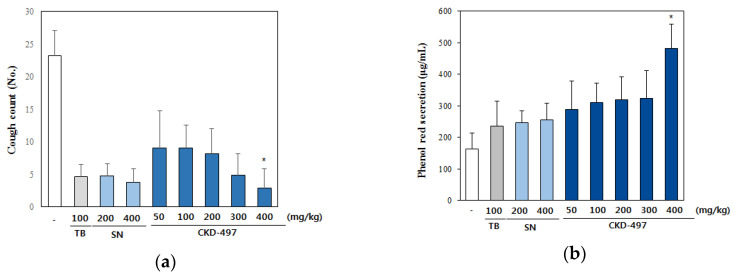
CKD-497 reduced the level of cough and increased expectoration of phlegm. (**a**) Spray of 5% citric acid induced cough after oral administration of CKD-497 in guinea pigs for 30 min. Then we counted the number of instances of coughing. (**b**) CKD-497 and positive controls were orally administered in mice for 30 min. Then, phenol red was injected in respiratory system for another 30 min. After euthanizing mice, respiratory system was extracted and washed. Optical density was measured with the liquid. Data are expressed as the mean ± SEM (*n* = 8). * *p* < 0.01 compared with Synatura 200 mg/kg group.

For our in vivo study, we determined the therapeutic potential of CKD-497 using guinea pigs and mice for cough inhibition and expectoration tests, respectively. At the tested doses, CKD-497 reduced cough frequency compared with the control group. In particular, CKD-497 at a dose of 400 mg/kg demonstrated significant inhibition of cough frequency compared to the positive controls SN and theobromine (Figure 5a). As shown in Figure 5b, the positive control (SN-treated groups) and CKD-497-treated groups increased tracheal phenol red secretion. At a dose of 400 mg/kg of CKD-497, phenol red output increased to 197% compared with the control group (Figure 5b).

### 2.6. The Effects of CKD-497 on Mucociliary Clearance In Vivo

**Figure 6:**
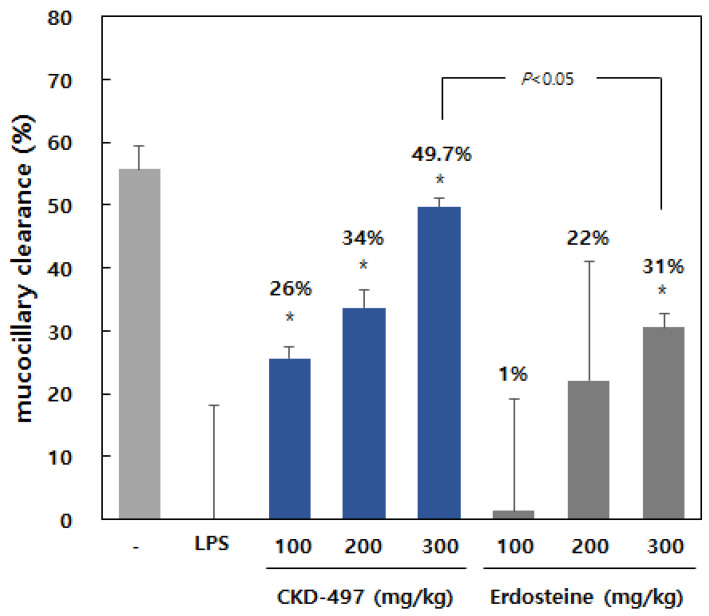
CKD-497 increased mucociliary clearance in rats. After euthanasia, rats were induced respiratory infection by LPS. After 3 h, CKD-497 or positive control was orally administrated and then 5% carbon solution was injected in bronchoalveolar lavage (BAL). We measured the optical density of the liquid extracted from BAL. Data are expressed as the mean ± SEM (*n* = 3–5). * *p* < 0.01 compared with vehicle.

Mucociliary clearance is a key factor in many chronic airway diseases. We also investigated the effect of CKD-497 on expectoration of phlegm using mucociliary clearance in the LPS-stimulated rats. CKD-497 significantly recovered the downregulated mucociliary clearance by LPS treatment in a dose-dependent manner. CKD-497 at 300 mg/kg induced more significant mucociliary clearance than erdosteine (positive control, Figure 6).

## 3. Discussion

This combination treatment of plant extracts may produce synergistic effects for specific biological responses of interest, or widen the therapeutic spectrum by summing different bioactivities originating from individual component plants, leading to good outcomes for complicated diseases [17]. Dysregulated cough and excess phlegm formation are associated with many factors like inflammatory mediators, irritants, bacteria, or virus [18,19]. Inflammatory mediators or cytokines like NO, IL-8, 12, 13, TNF-α, and TGF-β participate in forming pathological phlegm and provoking coughing. Hence, to some extent, the anti-inflammatory activities of plant extracts alleviated coughing and phlegm [20,21]. Atractylodis Rhizoma Alba and Fructus Schisandrae constitute the extract CKD-497 used in the present study. Atractylodis Rhizoma Alba was reported to inhibit inducible NO synthase [22] and inflammatory cytokines through mitogen-activated protein kinase (MAPK) and nuclear factor κB (NF-κB) [23,24]. Individual constituents of Fructus Schisandrae, schisandrin A, schisandrin, and gomisin N were reported to display anti-inflammatory effects [25,26,27]. Therefore, anti-inflammatory activity from CKD-497 could be expected, and in the current study, the production of NO and inflammatory cytokines (IL-8, IL-12, IL-13, TNF-α, and TGF-β1) was suppressed in LPS-stimulated macrophages. Cough is closely related to the expression of TRPV-1 in the epithelial and smooth muscle cells of the airway, and TRPV-1 receptor agonists such as capsaicin, acid, and arachidonic acid analogs induce acute coughing [16,28,29,30,31]. This receptor was also involved in the inflammatory response [32]. Under the influence of a TRPV-1 agonist, inflammatory cytokines could aggravate coughing [32,33]. In the TRPV-1-overexpressed BEAS-2B human bronchial epithelial cells treated by capsaicin, CKD-497 was found to downregulate inflammatory IL-8 and TNF-α releases and inhibit TRPV-1 expression. 

In the Republic of Korea, Synatura, composed of ivy leaf and Coptidis Rhizoma, used as one of the positive controls in the present study, is prescribed to treat symptoms including cough and phlegm due to acute upper respiratory tract infection and chronic bronchitis. CKD-497 activity was compared with Synatura. Thus, CKD-497 might exert suppression of coughing frequency in citric-acid-induced models (Figure 5). In addition, mucociliary clearance is one of the most important nonspecific defense mechanisms of the respiratory tract, and its impairment is a well-documented feature of chronic respiratory diseases [34,35,36]. In this regard, making phlegm mobile and less viscous or increasing mucociliary clearance are helpful for expectorating phlegm easily from the airway. CKD-497 loosened the viscous mucin significantly and enhanced expectoration ability and mucociliary clearance of phlegm (Figure 6), suggesting CKD-497 could ameliorate the symptoms of cough and facilitate expectoration of phlegm. 

In conclusion, CKD-497 was able to reduce the levels of inflammatory cytokines and TRPV-1 expression in vitro. CKD-497 could alleviate the cough symptoms and facilitate the clearance of phlegm. The in vitro and in vivo data, taken together, suggest that CKD-497 has great potential in controlling cough and phlegm.

## 4. Materials and Methods 

### 4.1. Material 

CKD-497, a new botanical drug candidate of the Chong Kun Dang (CKD) pharmaceutical company, consists of the roots of *Atractylodes japonica* Koidz and the fruits of *Schisandra chinensis* Baillon (5:1 weight ratio), obtained in the Jilin province in China in January 2017. These samples (root and fruit) were identified by emeritus professor Hyung Joon Chi, Seoul National University. The voucher specimen (CKD-BD-1701, CKD-BD-1702) was deposited at CKD research institute. Dried samples (total 1 kg) were extracted with 50% ethanol (EtOH) at room temperature for two days. Then, the extract solution was evaporated for dryness under reduced pressure after filtering with 2.5 μm papers (Waters, No. 5), yielding 290 g of CKD-497 in total. The reference compounds (6(*E*),12(*E*)-tetradecadiene-8,10-diyne-1,3-diol and schisandrin) were purchased for quantitative analysis from Wako Chemicals (Osaka, Japan).

### 4.2. Isolation of Reference Marker Compounds and High Pressure Liquid Chromatography (HPLC) Analysis

A portion (130 g) of crude extract was suspended in 1.2 L H_2_O and partitioned with organic solvents *n*-hexane, chloroform, and *n*-butanol, successively, using 1.2 L three times for each solvent, to produce the residues of *n*-hexane-soluble extract (8.5 g), chloroform-soluble (6.3 g), *n*-butanol-soluble extract (53.2 g), and water-soluble extract (72.6 g). The hexane-soluble extract (8.5 g, SH) was chromatographed with a silica column chromatography (5 × 15 cm, 100 g) using gradients of increasing polarity with hexane: ethyl acetate (100:0, 20:1, 10:1, 5:1, and 2:1) as solvents, and then fractionated into five sub-fractions (SH-1 to SH-5). The SH-2 fraction (160.1 mg) was subjected to HPLC separation with MeCN–H_2_O (40:60), 3.0 mL/min, by isocratic elution for 30 min and then 100% MeCN for 5 min, to produce compound atractylenolide I (t_R_ 26.14 min, 3.1 mg) and gomisin N (t_R_ 29.02 min, 3.7 mg). The SH-4 fraction (130.7 mg) was subjected to HPLC separation with MeCN–H_2_O (30:70 to 50:50), 3.0 mL/min, by gradient elution for 15 min, for 40 min, and then 100% MeCN for 5 min to produce compounds schisandrin (t_R_ 17.21 min, 6.6 mg) and gomisin A (t_R_ 23.15 min, 6.3 mg). The SH-6 fraction (56.2 mg) was subjected to HPLC separation with MeCN–H_2_O (35:65), 1.0 mL/min, by gradient elution for 20 min and then 100% MeCN for 5 min, to produce 6(*E*),12(*E*)-tetradecadiene-8,10-diyne-1,3-diol (t_R_ 17.75 min, 2.8 mg). The structures of isolates were confirmed by comparing the measured ^1^H and ^13^C NMR of the isolates (Figures S2–S11) with the published values [37,38,39,40,41]. 

Initial HPLC-UV analysis for CKD-497 extract was conducted using five isolated compounds (schisandrin, gomisin A, atractylenolide I, gomisin N, 6(*E*),12(*E*)-tetradecadiene-8,10-diyne-1,3-diol) and three compounds (schisandrin A, schisandrin C, and γ-schisandrin) obtained from the in-house chemical library, and the results are shown in Figure 1.

Further quantitative analysis of CKD-497 for two main compounds, 6(*E*),12(*E*)-tetradecadiene-8,10-diyne-1,3-diol and schisandrin, was conducted by using HPLC (Waters e2695, MA, USA) equipped with photodiode array detectors (Waters PDA, 2998) at 260 nm. A reversed-phase Kromasil 100-5C18 column (5.0 μm, 4.6 µm × 252 mm, NY, USA) was used. The gradient mixture of water (A) and acetonitrile (B) was set up as follows: a gradient elution (A:B = 70:30→50:50) from 0 to 8 min, a gradient elution (A:B = 50:50→20:80) from 8 to 20 min, an isocratic elution (A:B = 70:30) from 20 min to 25 min, a gradient elution (A:B = 70:30) from 25 to 26 min, and then an isocratic elution ((A:B = 70:30).

### 4.3. Cell Culture and Chemicals 

The Raw 264.7 murine macrophage cell line was purchased from ATCC (TIB-71) and maintained in Dulbecco′s Modified Eagle′s (DMEM) high glucose (Gibco, Grand Island, NY, USA) with 10% heat-inactivated fetal bovine serum (FBS, Gibco^®^), 300 mg/L L-glutamine, 25 mM HEPES, and 25 mM NaHCO_3_ (Gibco) in a 5% humidified CO_2_ incubator at 37 °C (Thermo Scientific, Waltham, MA, USA). Synatura was purchased from Seaha Healthcare (Korea). Theobromine, ambroxol, and lipopolysaccharide (LPS) were purchased from Sigma-Aldrich (St. Louis, MO, USA).

The BEAS-2B normal human bronchial epithelial cell line was purchased from Lonza (Basel, Switzerland). The flasks were pre-coated with 0.01 mg/mL fibronectin, 0.03 mg/mL bovine collagen type I, and 0.01 mg/mL bovine serum albumin (BSA) in Bronchial Epithelial Cell Growth Medium (BEGM, Lonza, Basel, Switzerland) for 4 h to overnight. After coating, the container was washed with sterilized phosphate-buffered saline (PBS) twice.

### 4.4. TRPV-1 Overexpressed BEAS-2B Human Bronchial Epithelial Cell Line

Human RNA was extracted using TRI reagent (Sigma, MO, USA) and cDNA was synthesized with a high-capacity cDNA reverse transcription kit (Applied Biosystems, Foster City, CA, USA) in accordance with the manufacturers’ protocol. PCR was conducted 30 cycles in conditions of 10 s at 98 °C; 30 s at 62 °C; 1 min at 72 °C (Premix Takara, Kusatsu, Japan), and this product was inserted into pcDNA 3.1D/V5-His-TOPO vector (Invitrogen, Carlsbad, CA, USA). After this complete vector was transformed into one-shot TOP10 competent cells, the colony was selected by ampicillin. To make the TRPV-1-overexpressed BEAS-2B cell line, 2 × 10^4^ cells were plated in one of the 6-well plates for 24 h. Then the pcDNA 3.1D/V5-His-TOPO-TRPV-1 construct was transfected with lipofectamine 2000 (Invitrogen) and selected by using F418 200 μg/mL, which generated in the stable transfected cell line.

### 4.5. Measurement of Nitric Oxide (NO) and Cytokine Production

To determine nitric oxide (NO) in Raw 264.7 cells, 2 × 10^6^ cells/mL were seeded in 24 wells. CKD-497 at 0, 25, 50, 100, and 200 µg/mL, and positive control SN at 200 µg/mL, were pre-treated for an hour. Then, inflammation was induced by LPS (1 μg/mL) for 24 h. The media were harvested and we detected NO assay and cytokine levels. To perform the NO assay, NaNO_2_ was used as the standard. After adding 100 μL of Griess reagent (Sigma) to 100 μL of harvested media, it was incubated for 5 min in 96-well plates. The optical density was measured at 570 nm using a spectrophotometer (SpectraMax M2, Molecular devices, USA). The levels of IL-8 (BT-laboratory, Birmingham, U.K.), IL-12, IL-13, TNF-α, and TGF-β1 (eBIOSCIENCE, Thermo Fisher Scientific, Waltham, MA, USA) were detected by an enzyme-linked immunosorbent assay (ELISA) kit from each company according to manufacturers’ instructions.

### 4.6. Viscosity Test

The viscosity solution consisted of 15% mucin solution in 30 mM Tris buffer, pH 7.4. Vehicle, CKD-497, *N*-acetylcysteine, or erdosteine (10 mg/mL) was treated by stirring for an hour at 37 °C. Viscosity (mPa·s) was measured using a microrheometer (MCR 302, Anton Paar, Graz, Austria).

### 4.7. Animals 

The animal experiments were performed according to the U.S. National Institutes of Health (NIH) Guidelines for the Care and Use of Laboratory Animals with the Institutional Animal Care and Use Committee (P150041, P150042) of CKD in Korea. Their house was maintained at 20.8–24.7 °C and 47.8–56.6% humidity under a 12 h light/dark cycle with free access to food and tap water.

### 4.8. Analysis of Cough and Expectorant Activity In Vivo 

The method of Zhuang et al. [42] was used with some modification. To conduct the cough experiment, 72 guinea pigs (5 weeks old, 216–286 g) were bought from SamTako Bio Korea Inc. (Osan, Korea). Groups were divided into one control group, three positive control groups (200 and 400 mg/kg of Synatura and 100 mg/kg of theobromine), and five experimental groups (50, 100, 200, 300, 400 mg/kg of CKD-497). Each group contained 8 guinea pigs. After orally administered positive control or CKD-497, the instances of coughing were counted during administration of 5% citric acid gas to be inhaled using nebulizer in the cage.

For the expectorant test, a total of 72 institute of cancer research (ICR) mice (5 weeks old, 22–26.5 g) were purchased from Orient Bio (Orient Bio Inc., Seongnam, Korea). The vehicle, two positive controls (Synatura or ambroxol), and CKD-497 were orally administrated, and 30 min later, 5% phenol red in saline (0.1 mL/10 g) was also administered by intraperitoneal injection. Another 30 min later, mice were terminated with spinal dislocation, and then bronchial extraction was conducted. Phenol red was extracted in 1 mL saline during sonication, and optical density was measured at 558 nm in addition to 5% sodium bicarbonate solution. 

### 4.9. Mucociliary Clearance Test 

The method of Vander Top et al. [43] was used with some modification. A total of 39 Sprague Dawley (SD) rats (5 weeks old, 138–168 g) were purchased from Orient Bio (Orient Bio Inc., Seongnam, Korea) and stabilized for seven days. The control rats and lipopolysaccharides (LPS)-induced rats were in three groups. LPS injection (15 μg/100 μL) was conducted to induce respiratory infection in respiratory system after euthanasia with zoletil and xylazine. The experimental group (CKD-497) and positive control (erdosteine) were orally injected with 10 mL/kg of 100, 200, 300 mg/kg (4–5 rats per group) once 3 h after LPS injection. A 5% carbon solution was introduced to the respiratory system for 2 h, and then bronchoalveolar lavage (BAL) was harvested after termination with CO_2_ gas. The BAL solution was centrifuged for 10 min at 100 rpm and detected absorbance at 500 nm.

### 4.10. Statistics Analysis

Statistical analysis in all experiments was a two-way analysis of variance (ANOVA) with Graphpad (San Diego, CA, USA). The data were considered to be significant statistically if the probability had a value of 0.05 or less.

## Supplementary Materials

The following are available online, Figure S1: chemical structures of all compounds, Figures S2–S11: 1H and 13C NMR data of isolated compounds, Figures S12–S19: HPLC-UV chromatogram of all compounds, Figures S20–S24: MS spectrum of isolated compounds.
