# Supplementary material for: Standardized Extract of Atractylodis Rhizoma Alba and Fructus Schisandrae Ameliorates Coughing and Increases Expectoration of Phlegm
Source: Molecules. 2020 Jul 4;25(13):3064. doi: 10.3390/molecules25133064 (PMC7411911; doi:10.3390/molecules25133064)
Supplement: Supplementary file 1 [file molecules-25-03064-s001.pdf]

Supplementary materials

Standardized extract of Atractylodis rhizoma alba and Schisandrae fructus ameliorates coughing and increases expectoration of phlegm

Hee-Sung Chae[1], Sun Young Kim[2], Pisey Pel[1], Jungmoo Huh[1], Sun-Woo Joo[2], Yun Young Lim[2], Shin Jung Park[2], Jong Lae Lim[2] and Young-Won Chin[1,*]

[1] College of Pharmacy and Research Institute of Pharmaceutical Sciences, Seoul National University, 1, Gwanak-ro, Gwanak-gu, Seoul 08826, Republic of Korea; ywchin@snu.ac.kr, chaeheesung83@gmail.com, jmhuh112@gmail.com

[2] Department of Botanical Drug, Chong Kun Dang (CKD) pharm research Institute, Yongin-si, Gyeonggi-do, and Republic of Korea; syk@ckdpharm.com, sunwoo@ckdpharm.com, yylim@ckdpharm.com, parksj@ckdpharm.com, jllim@ckdpharm.com

* Correspondence: ywchin@snu.ac.kr; Tel.: +82-2-880-7859 (Y.W. Chin.)

Figure S1. Chemical structure of all compounds

Figure S2. ^{1}H-NMR (500 MHz, $CDCl_3$) spectrum of atractylenolide I

Figure S3. ^{13}C-NMR (125 MHz, $CDCl_3$) spectrum of atractylenolide I

Figure S4. ^{1}H-NMR (400 MHz, $CDCl_3$) spectrum of gomisin N

Figure S5. ^{13}C-NMR (100 MHz, $CDCl_3$) spectrum of gomisin N

Figure S6. ^{1}H-NMR (400 MHz, $CDCl_3$) spectrum of schisandrin

Figure S7. ^{13}C-NMR (100 MHz, $CDCl_3$) spectrum of schisandrin

Figure S8. ^{1}H-NMR (400 MHz, $CDCl_3$) spectrum of gomisin A

Figure S9. ^{13}C-NMR (100 MHz, $CDCl_3$) spectrum of gomisin A

Figure S10. ^{1}H-NMR (500 MHz, DMSO-d_6) spectrum of 6(*E*),12(*E*)-tetradecadiene-8,10-diyne-1,3-diol

Figure S11. ^{13}C-NMR (125 MHz, DMSO-d_6) spectrum of 6(*E*),12(*E*)-tetradecadiene-8,10-diyne-1,3-diol

Figure S12. HPLC-UV chromatogram of 6(*E*),12(*E*)-tetradecadiene-8,10-diyne-1,3-diol

Figure S13. HPLC-UV chromatogram of schisandrin

Figure S14. HPLC-UV chromatogram of gomisin A

Figure S15. HPLC-UV chromatogram of atractylenolide Ⅰ

Figure S16. HPLC-UV chromatogram of schisandrin A

Figure S17. HPLC-UV chromatogram of gomisin N

Figure S18. HPLC-UV chromatogram of γ-schisandrin

Figure S19. HPLC-UV chromatogram of schisandrin C

Figure S20. MS spectrum of atractylenolide Ⅰ

Figure S21. MS spectrum of gomisin N

Figure S22. MS spectrum of schisandrin

Figure S23. MS spectrum of gomisin A

Figure S24. MS spectrum of spectrum of 6(*E*),12(*E*)-tetradecadiene-8,10-diyne-1,3-diol

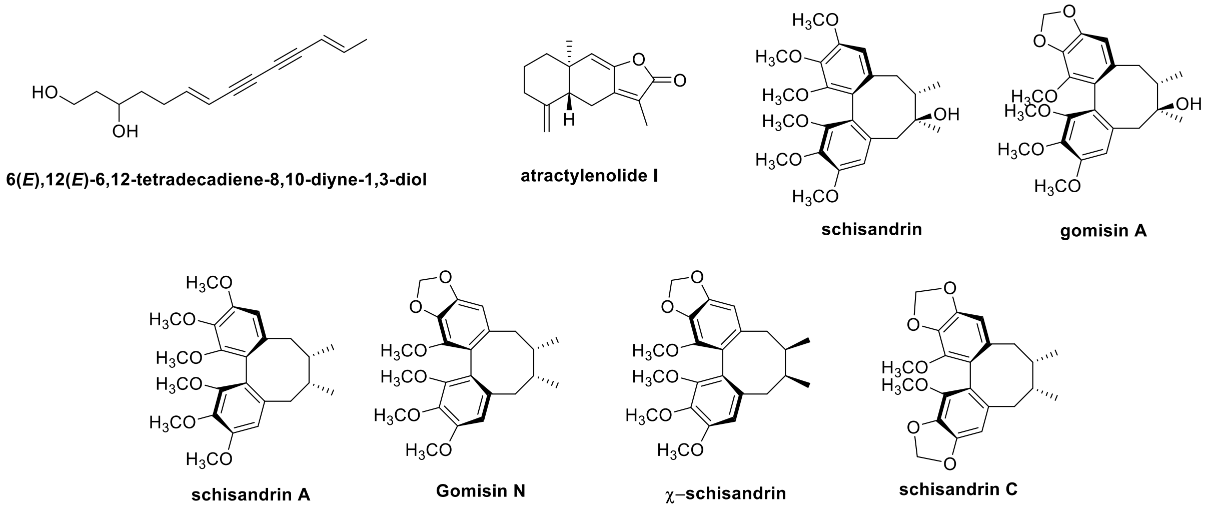

Figure S1. Chemical structures of all compounds

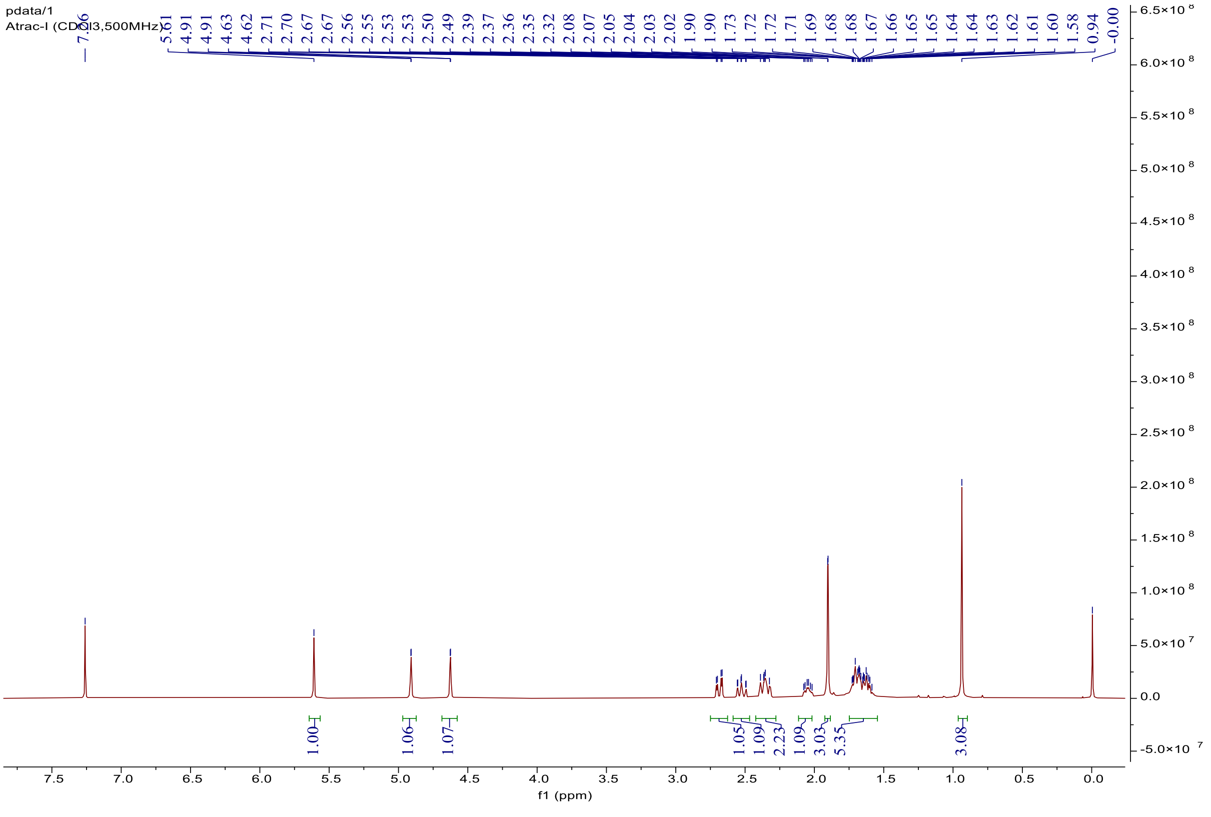

Figure S2. ^{1}H-NMR (500 MHz, $CDCl_3$) spectrum of atractylenolide I

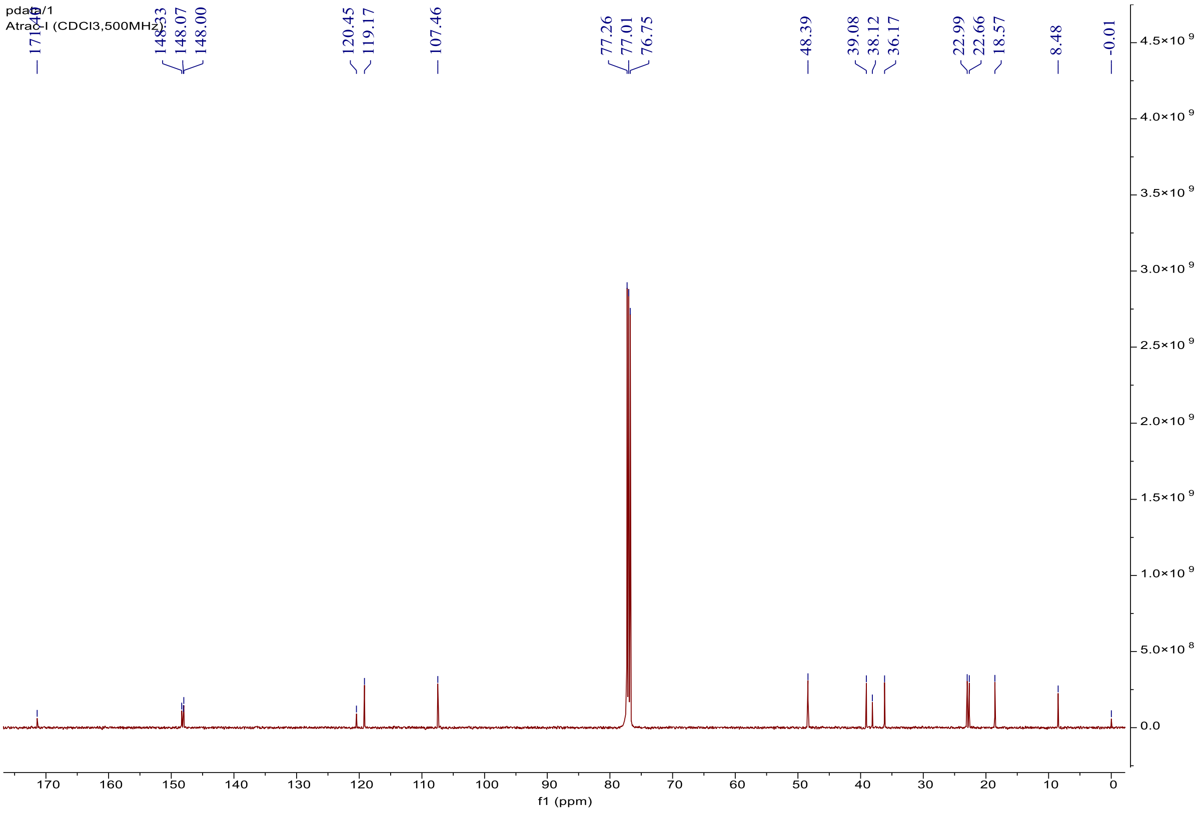

Figure S3. ^{13}C-NMR (125 MHz, $CDCl_3$) spectrum of atractylenolide I

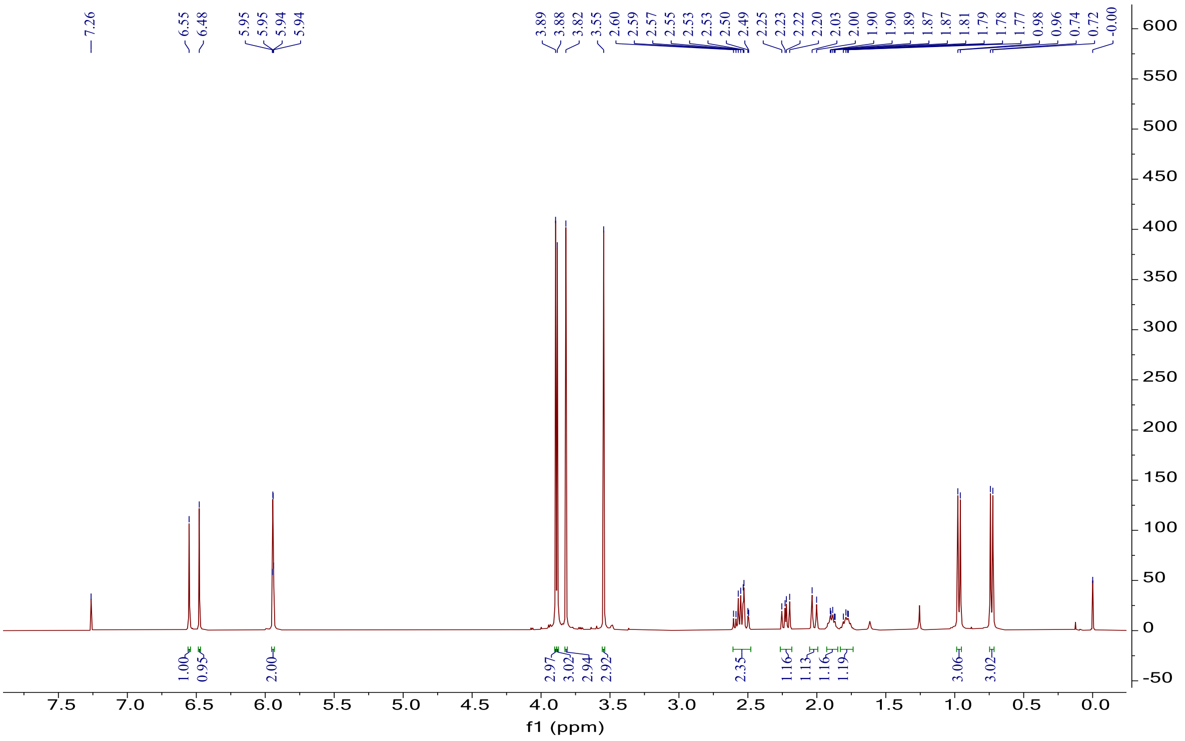

Figure S4. ^{1}H-NMR (400 MHz, $CDCl_3$) spectrum of gomisin N

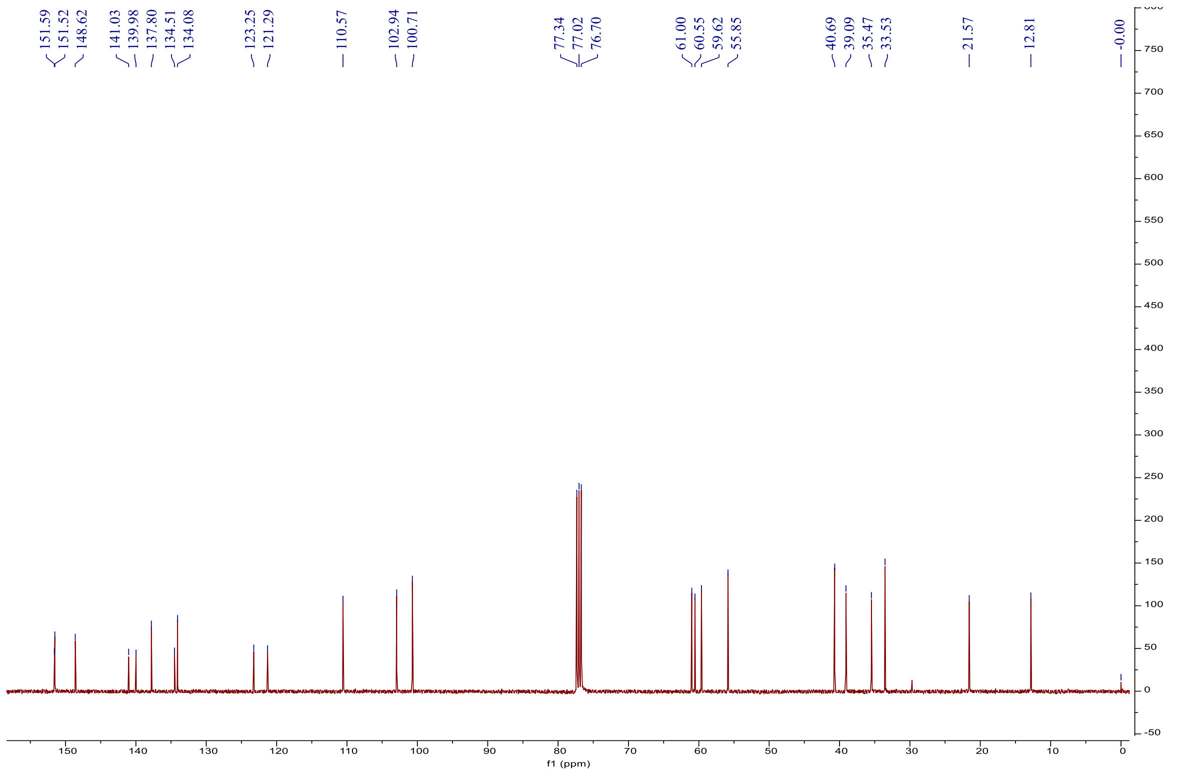

Figure S5. ^{13}C-NMR (100 MHz, $CDCl_3$) spectrum of gomisin N

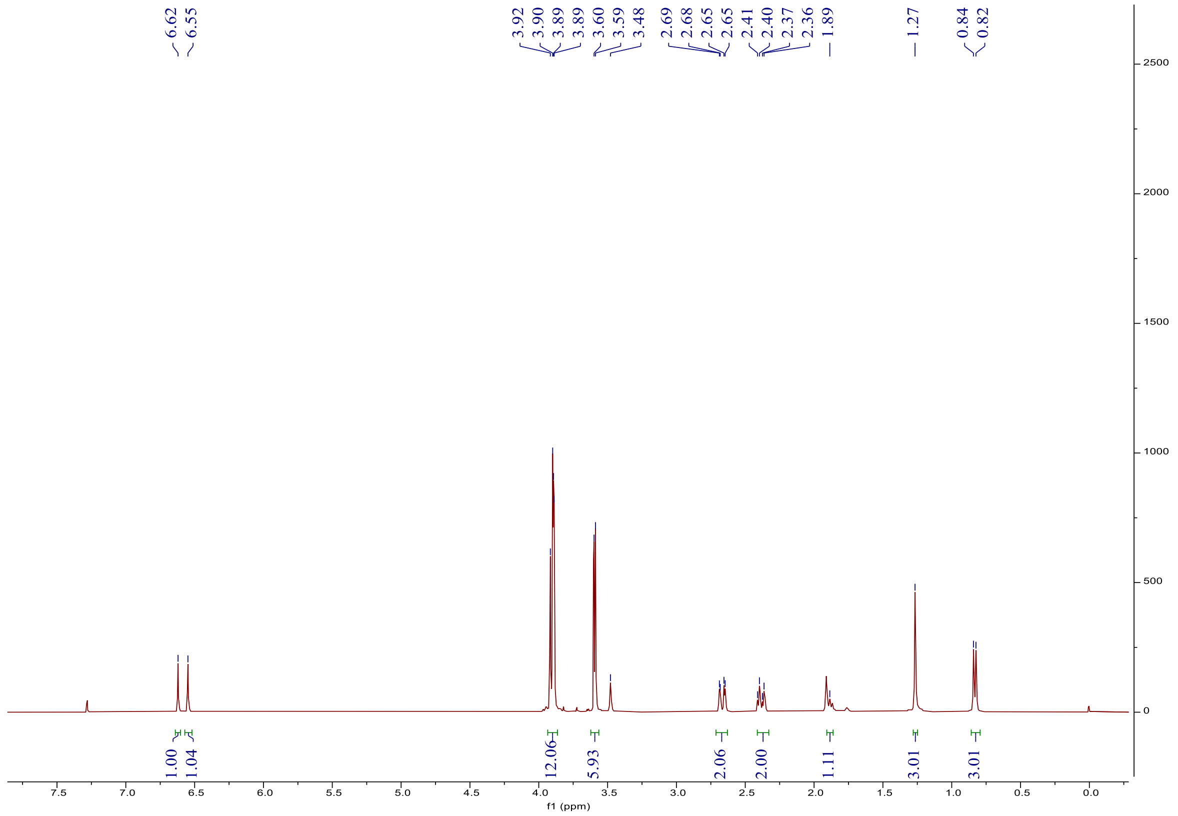

Figure S6. ^{1}H-NMR (400 MHz, $CDCl_3$) spectrum of schisandrin

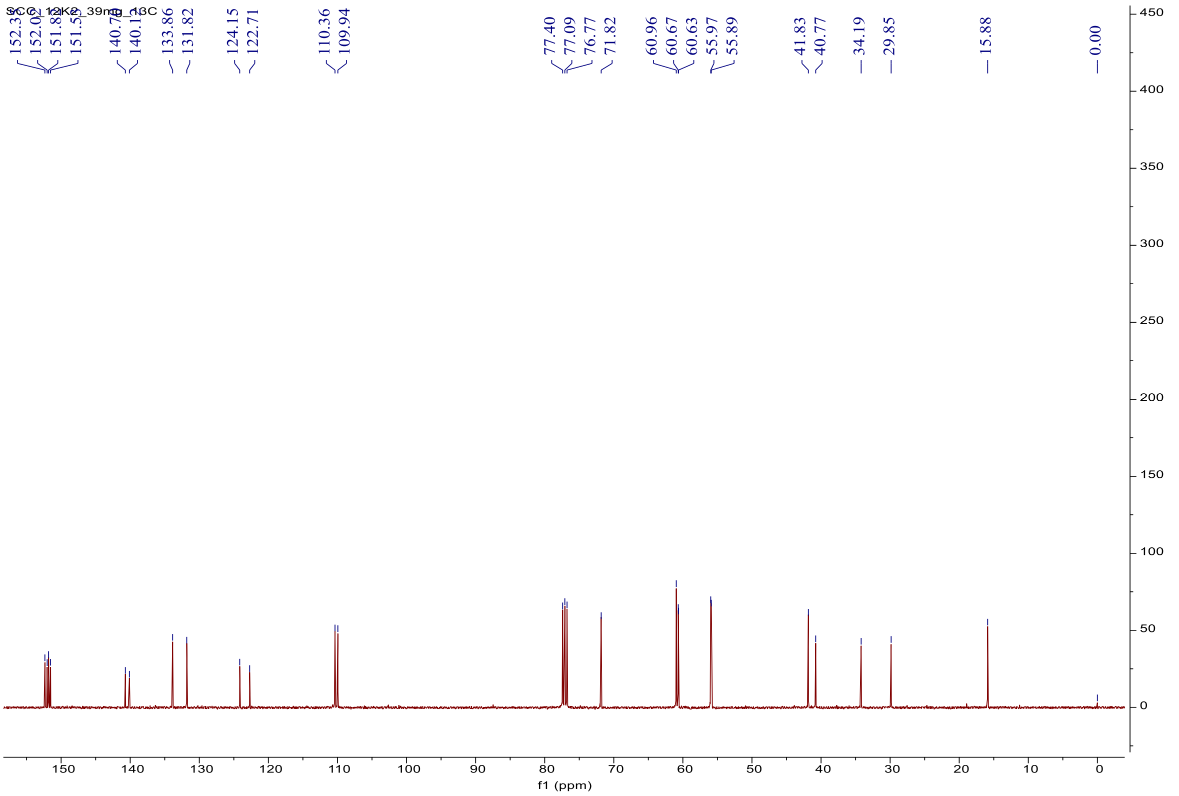

Figure S7. ^{13}C-NMR (100 MHz, $CDCl_3$) spectrum of schisandrin

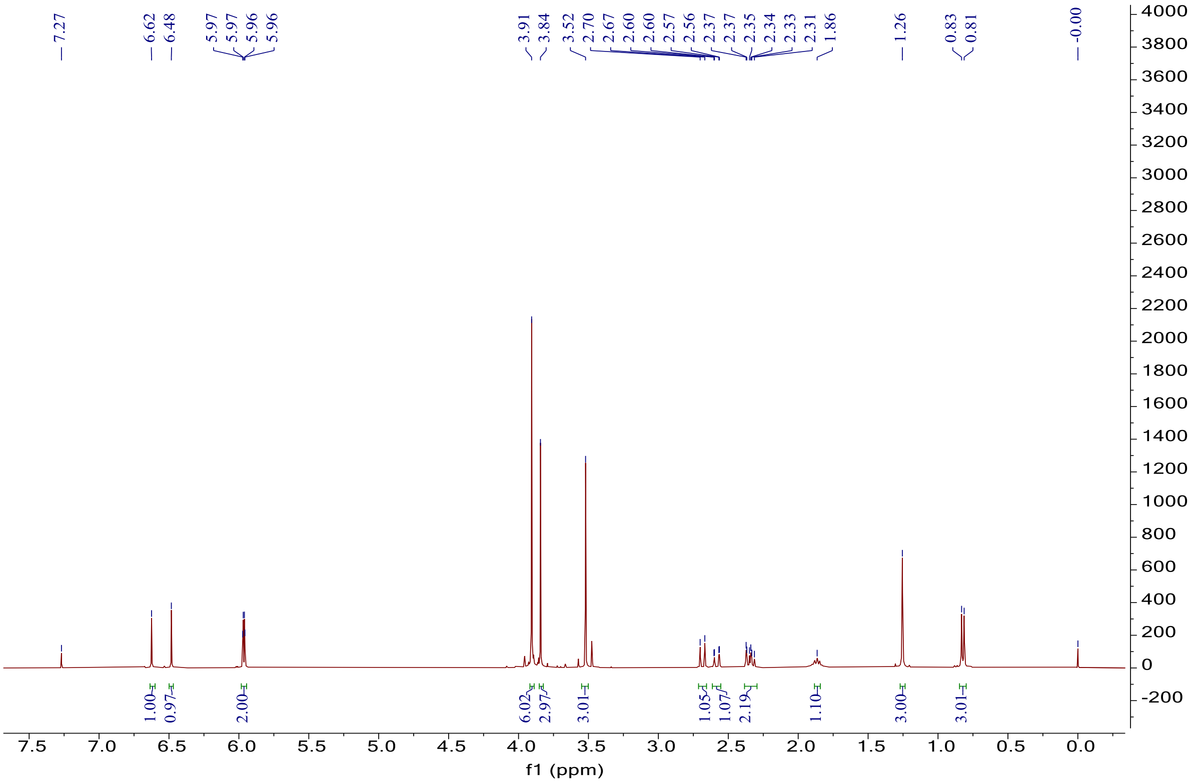

Figure S8. ^{1}H-NMR (400 MHz, $CDCl_3$) spectrum of gomisin A

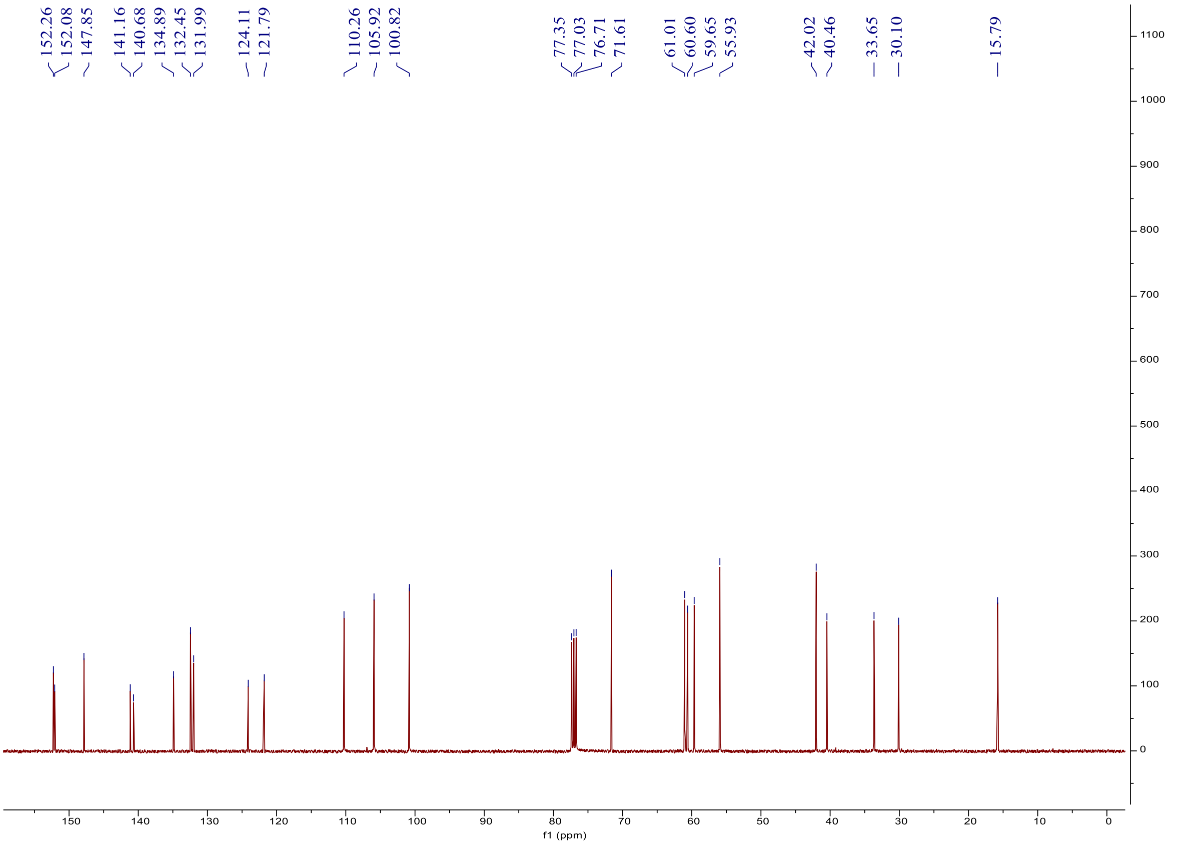

Figure S9. ^{13}C-NMR (100 MHz, $CDCl_3$) spectrum of gomisin A

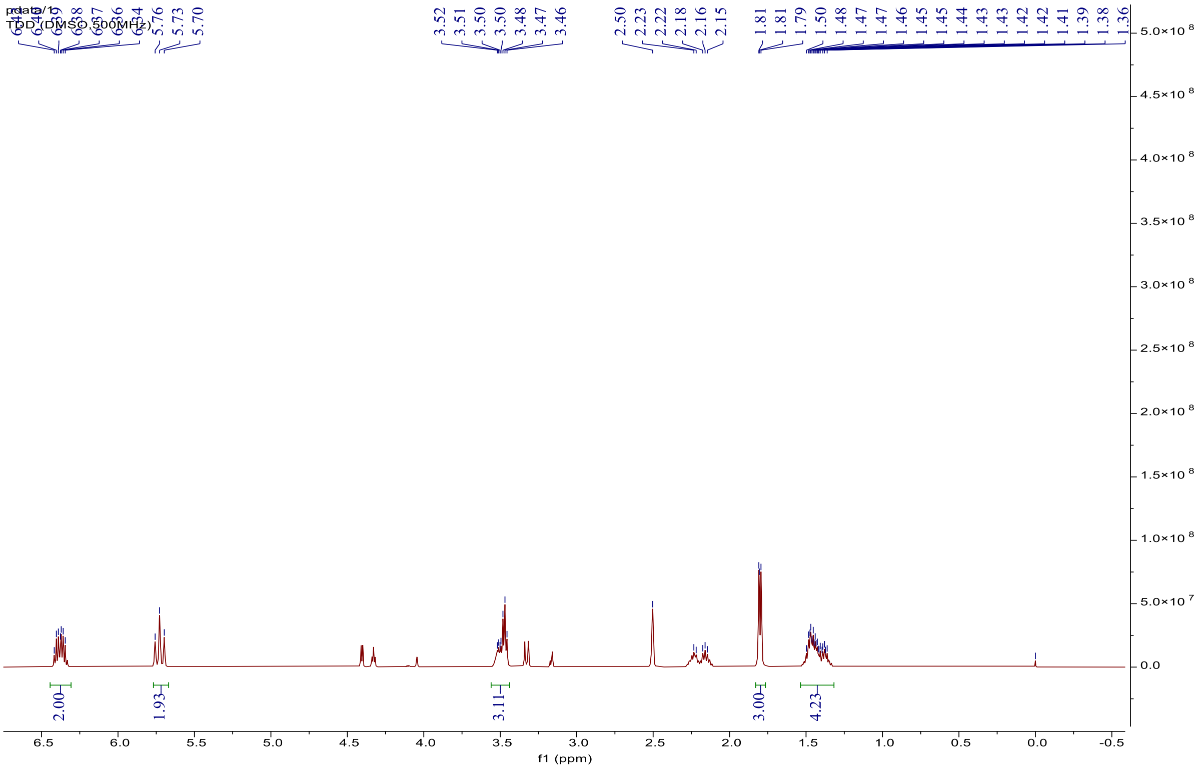

Figure S10. ^{1}H-NMR (500 MHz, DMSO-d_6) spectrum of 6(*E*),12(*E*)-tetradecadiene-8,10-diyne-1,3-diol

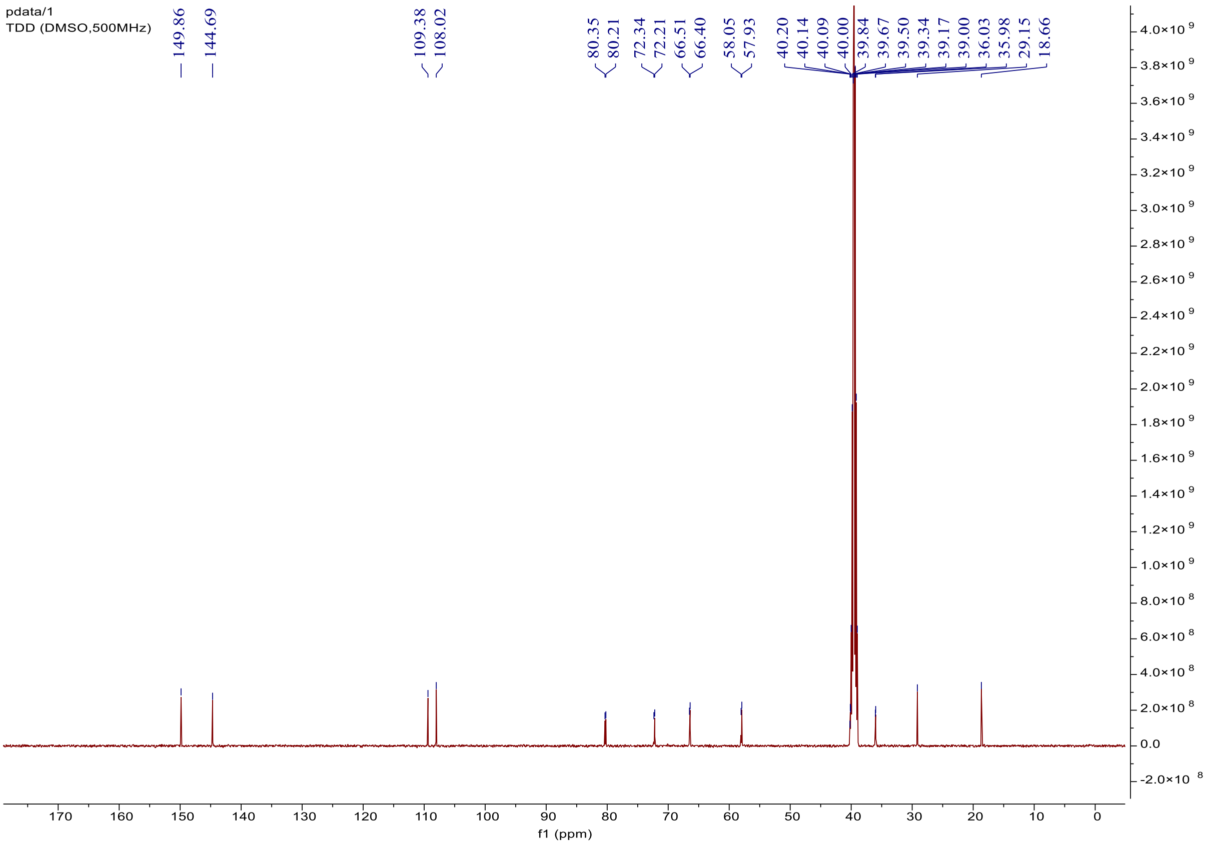

Figure S11. ^{13}C-NMR (125 MHz, DMSO-d_6) spectrum of 6(*E*),12(*E*)-tetradecadiene-8,10-diyne-1,3-diol

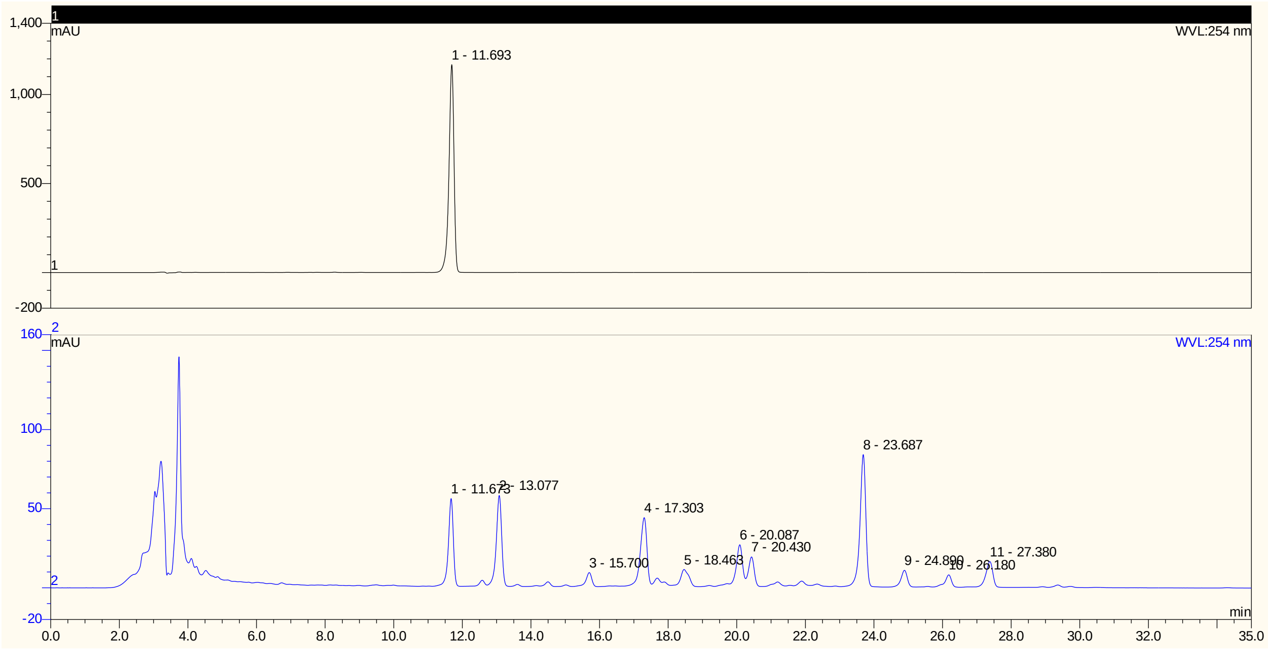

Figure S12. HPLC-UV chromatogram of 6(*E*),12(*E*)-tetradecadiene-8,10-diyne-1,3-diol

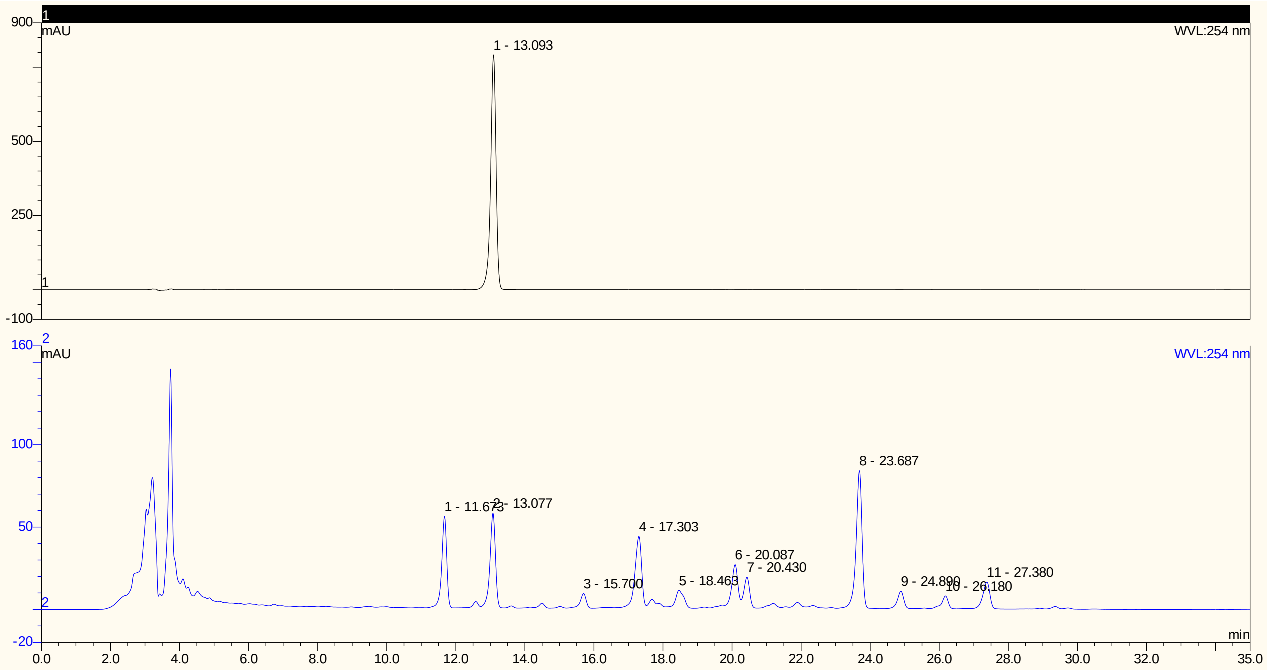

Figure S13. HPLC-UV chromatogram of schisandrin

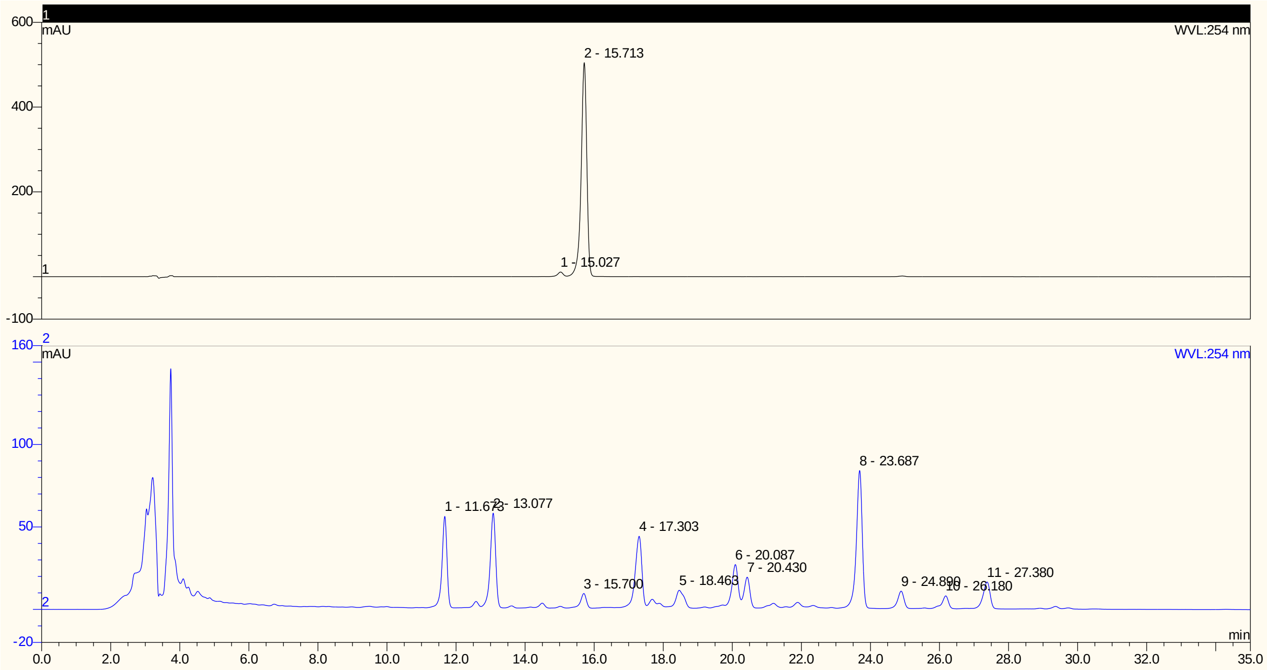

Figure S14. HPLC-UV chromatogram of gomisin A

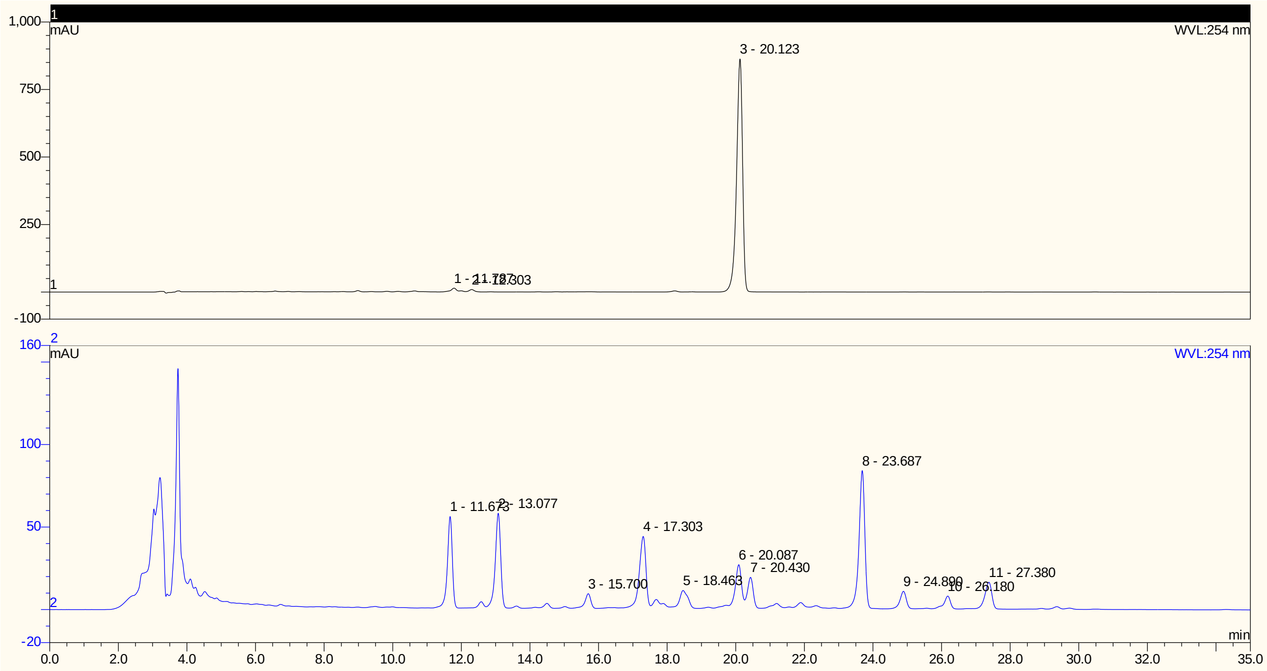

Figure S15. HPLC-UV chromatogram of atractylenolide Ⅰ

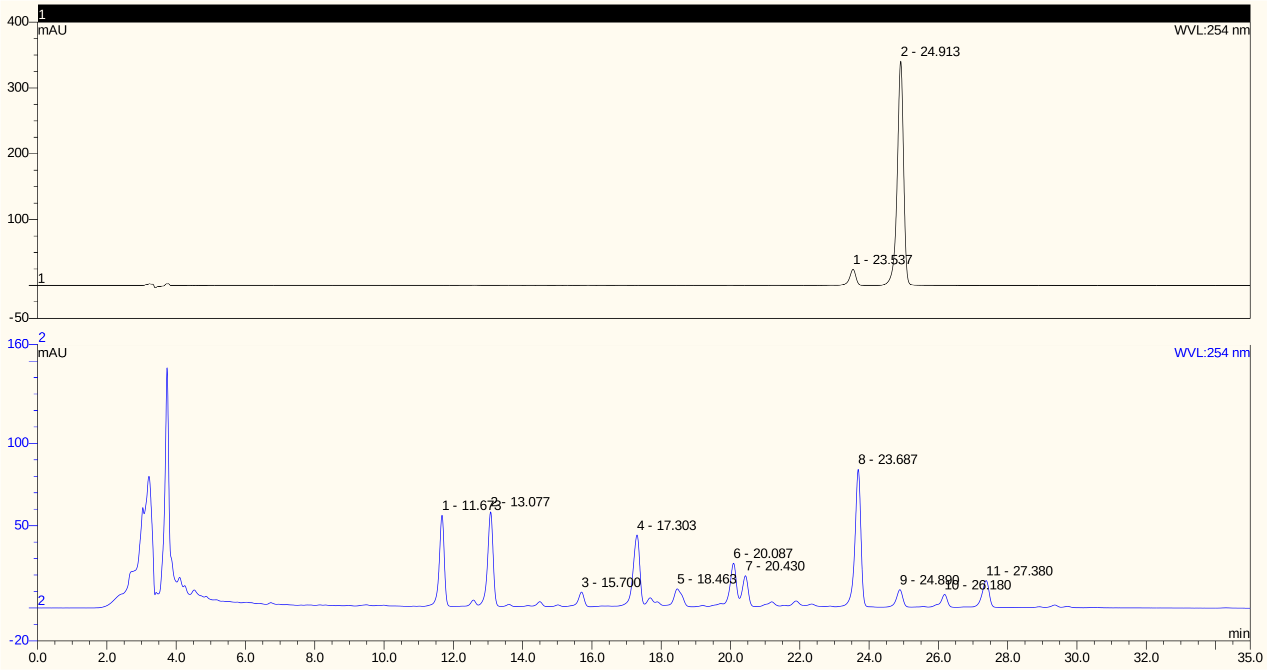

Figure S16. HPLC-UV chromatogram of schisandrin A

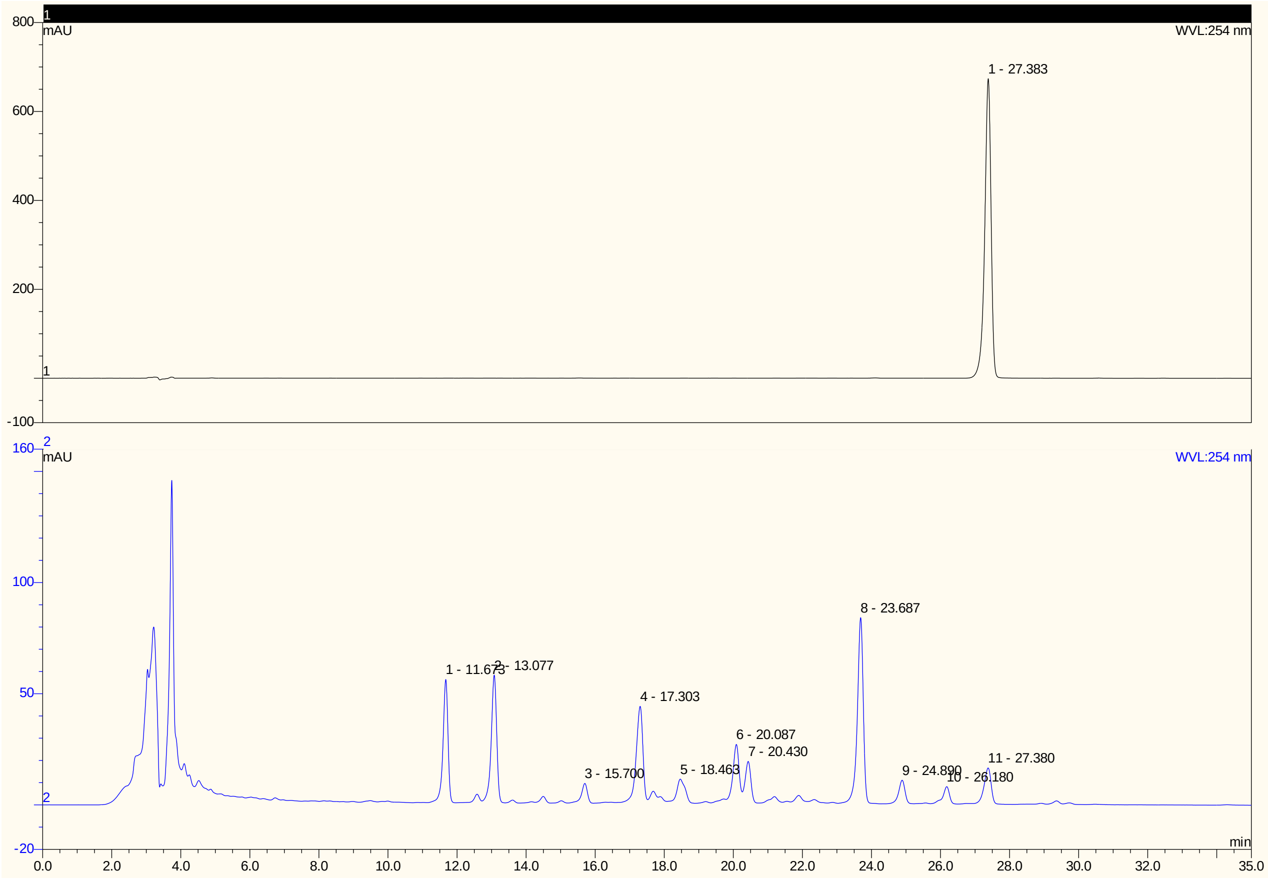

Figure S17. HPLC-UV chromatogram of gomisin N

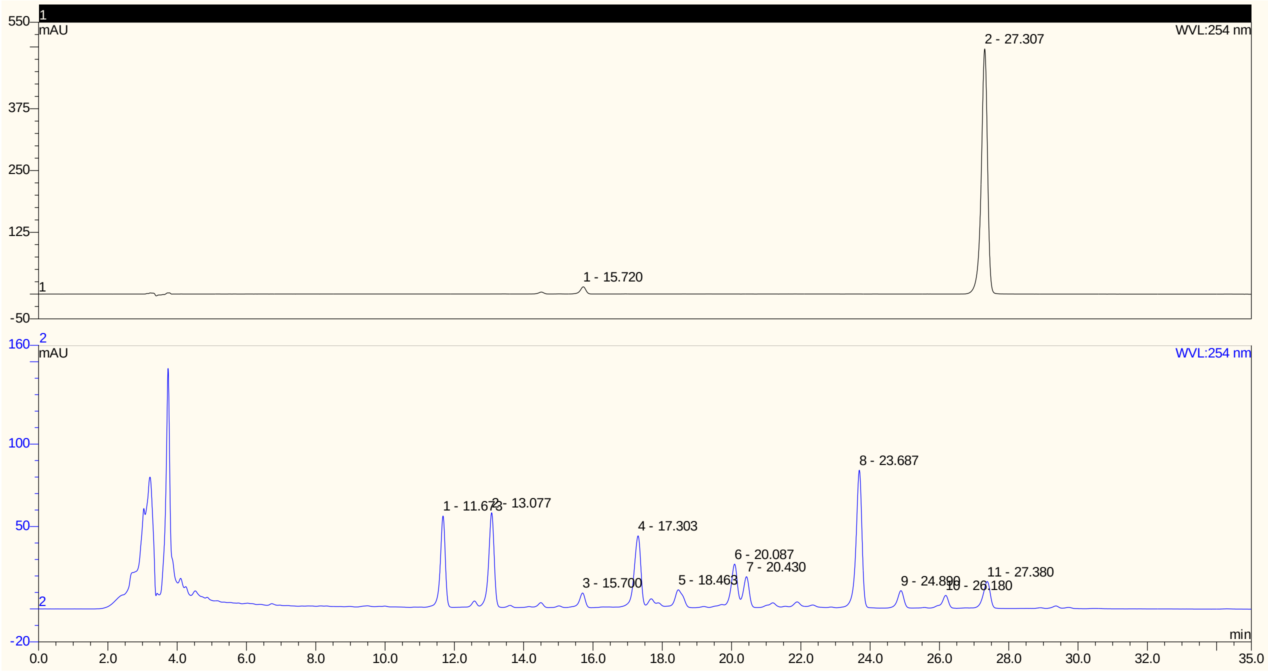

Figure S18. HPLC-UV chromatogram of γ-schisandrin

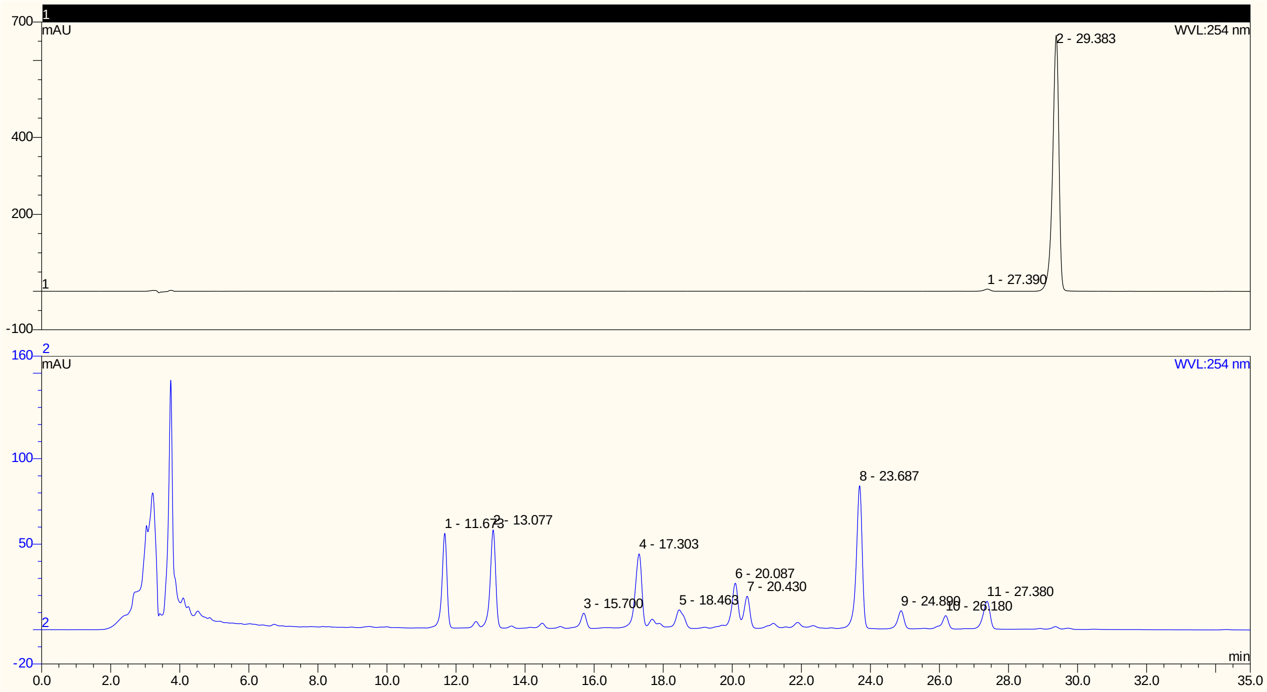

Figure S19. HPLC-UV chromatogram of schisandrin C

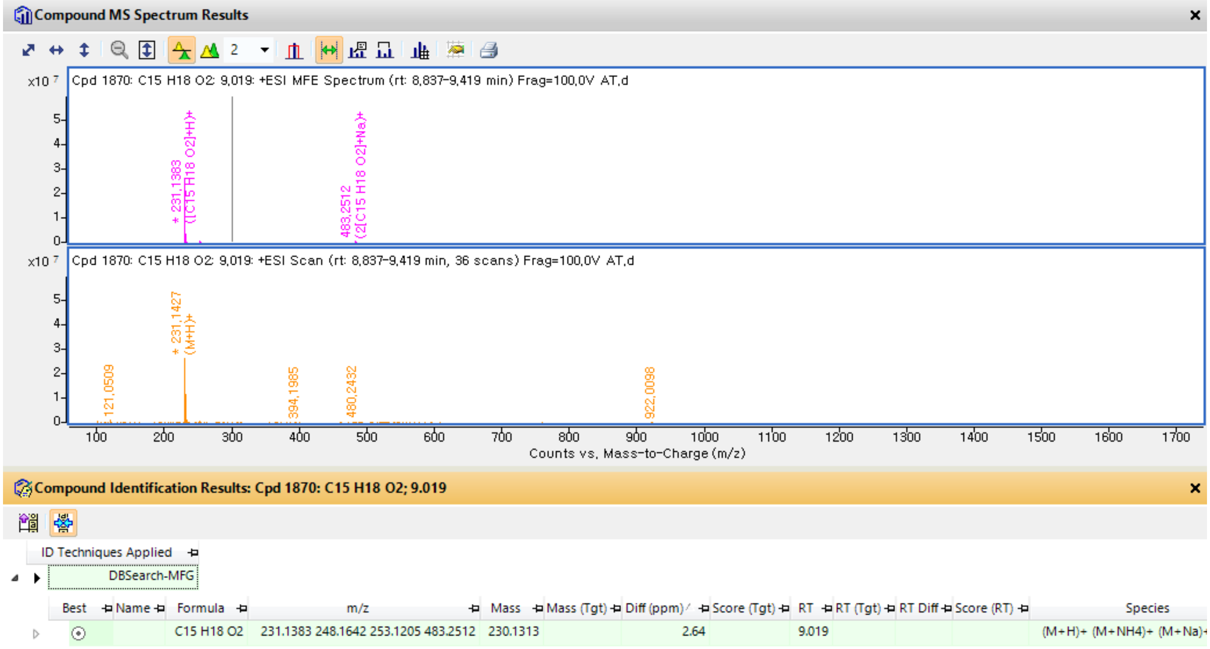

Figure S20. MS spectrum of atractylenolide Ⅰ

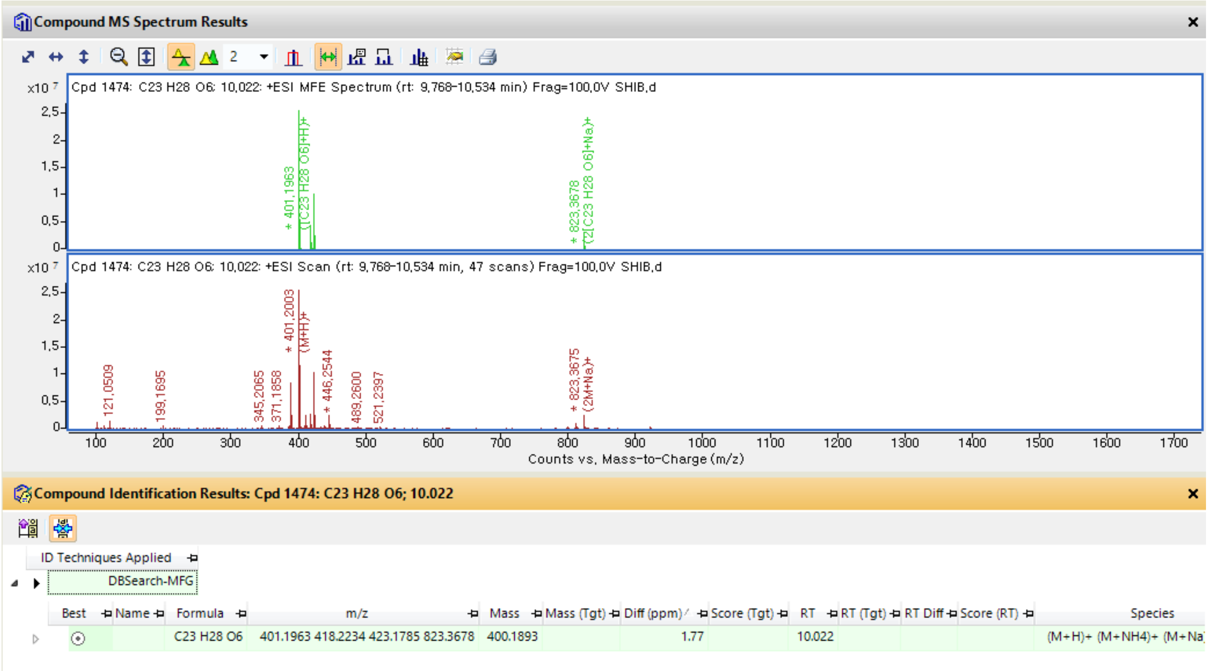

Figure S21. MS spectrum of gomisin N

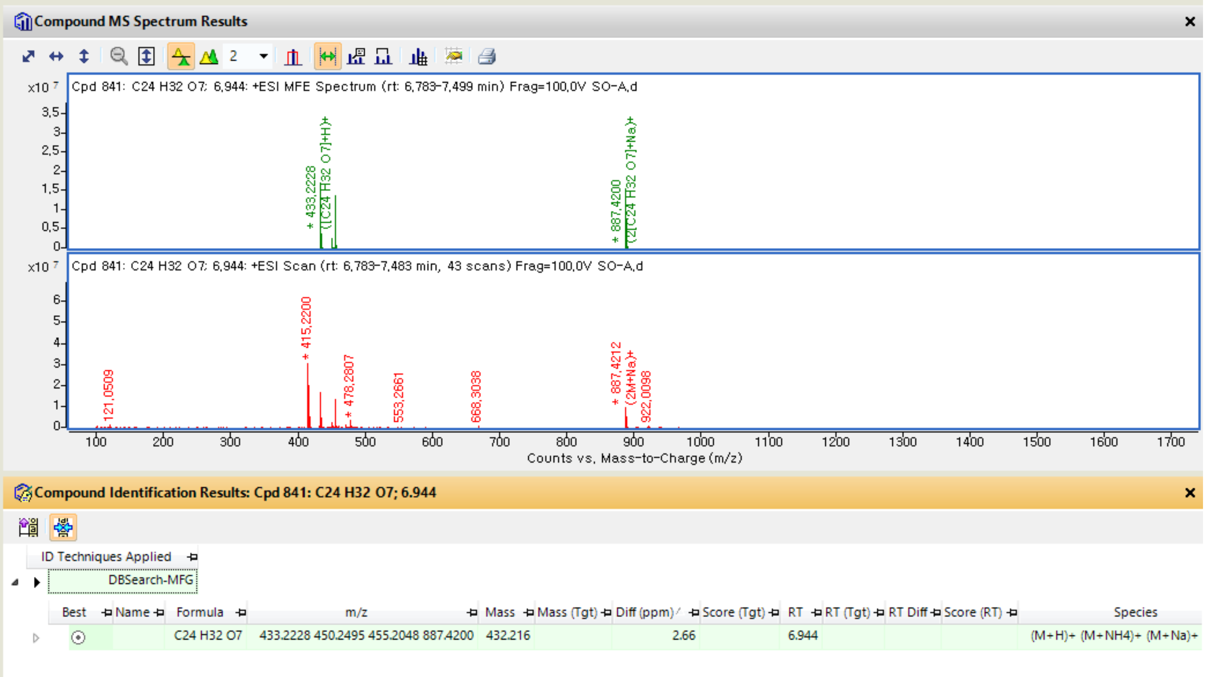

Figure S22. MS spectrum of schisandrin

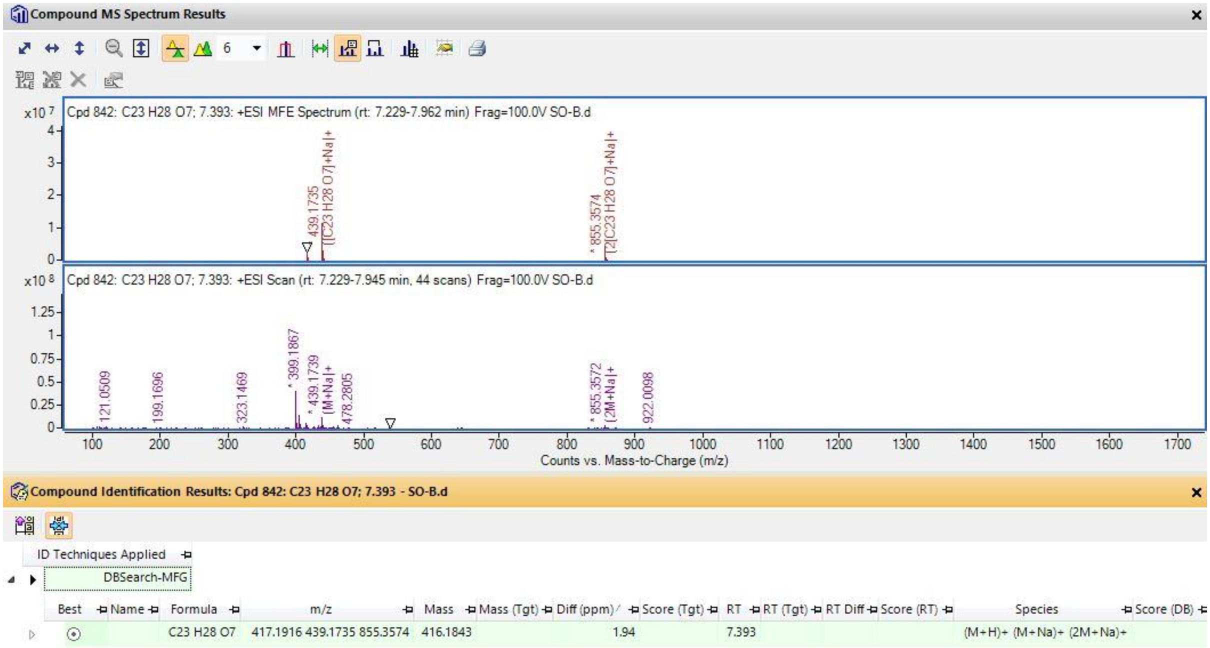

Figure S23. MS spectrum of gomisin A

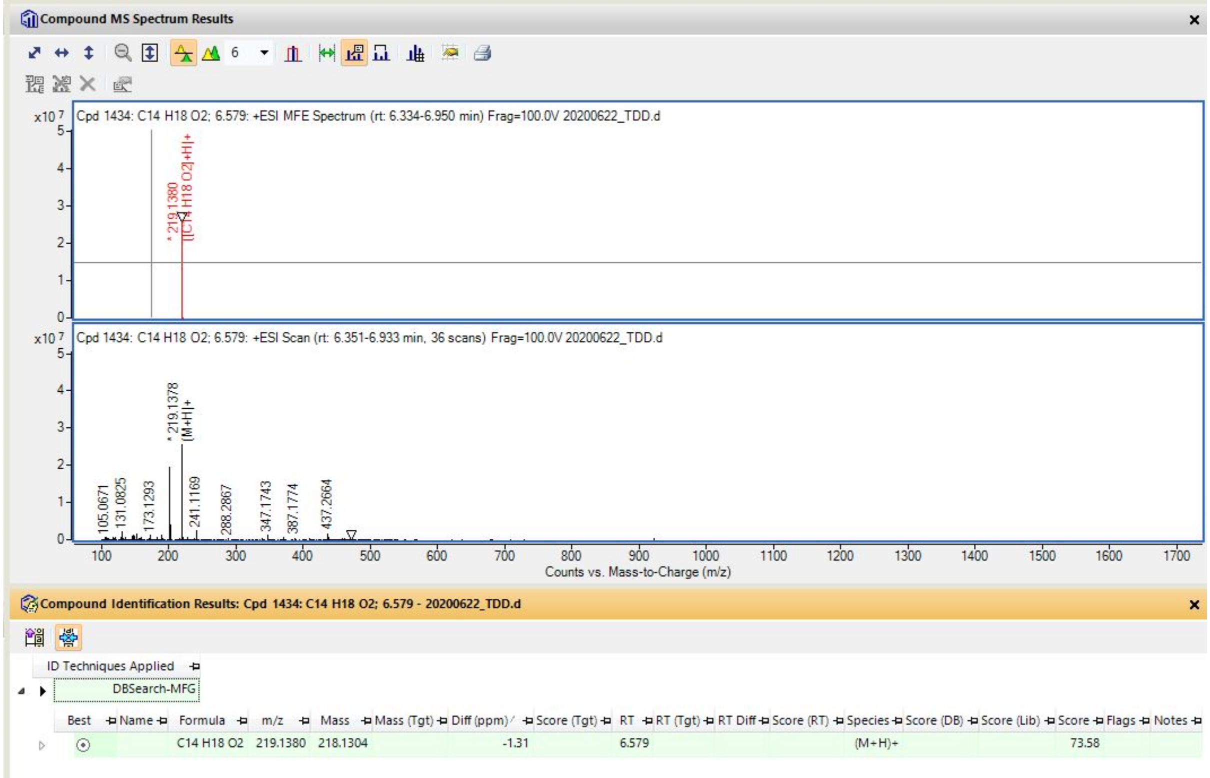

Figure S24. MS spectrum of spectrum of 6(*E*),12(*E*)-tetradecadiene-8,10-diyne-1,3-diol
